# Roles of MXenes in biomedical applications: recent developments and prospects

**DOI:** 10.1186/s12951-023-01809-2

**Published:** 2023-03-02

**Authors:** Hui Li, Rangrang Fan, Bingwen Zou, Jiazhen Yan, Qiwu Shi, Gang Guo

**Affiliations:** 1 State Key Laboratory of Biotherapy and Cancer Center, West China Hospital, Sichuan University, Chengdu, 610041 China; 2 School of Mechanical Engineering, Sichuan University, Chengdu, 610065 China; 3 College of Materials Science and Engineering, Sichuan University, Chengdu, 610065 Sichuan China

**Keywords:** MXenes, Synthesis, Biosensors, Diagnosis, Immunotherapy, Wearable device

## Abstract

....With the development of nanomedical technology, the application of various novel nanomaterials in the biomedical field has been greatly developed in recent years. MXenes, which are new inorganic nanomaterials with ultrathin atomic thickness, consist of layered transition metal carbides and nitrides or carbonitrides and have the general structural formula M_n+1_X_n_T_x_ (n = 1–3). Based on the unique structural features of MXenes, such as ultrathin atomic thickness and high specific surface area, and their excellent physicochemical properties, such as high photothermal conversion efficiency and antibacterial properties, MXenes have been widely applied in the biomedical field. This review systematically summarizes the application of MXene-based materials in biomedicine. The first section is a brief summary of their synthesis methods and surface modification strategies, which is followed by a focused overview and analysis of MXenes applications in biosensors, diagnosis, therapy, antibacterial agents, and implants, among other areas. We also review two popular research areas: wearable devices and immunotherapy. Finally, the difficulties and research progress in the clinical translation of MXene-based materials in biomedical applications are briefly discussed.

**Graphical Abstract:**

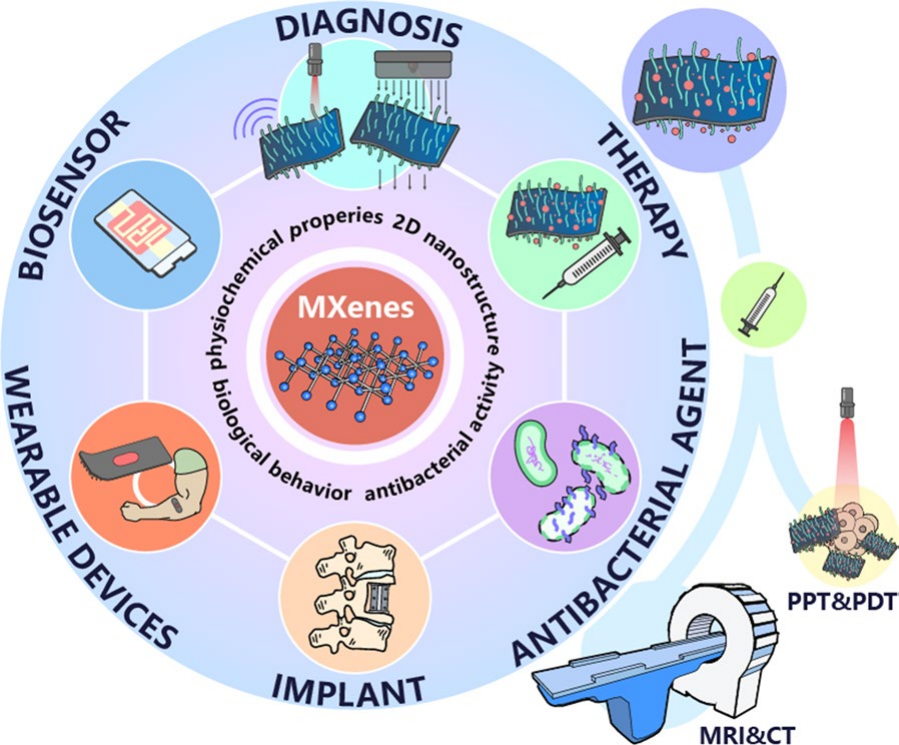

## Introduction

With the development of biomedicine and nanobiotechnology, diverse novel inorganic nanosystems have been generated, which enable multiple theranostic modalities, such as synergistic therapy and multimodal imaging, to be offered as potential alternatives in combating various diseases, especially cancer. Recently, a variety of two-dimensional (2D) nanomaterials, which are a subtype of nanomaterials with ultrathin layer-structured topology, have attracted great interest, including graphene and its derivatives, transition metal dichalcogenides (TMDCs), transition metal oxides transition metal oxides (TMOs), and transition metal carbides (MXenes). Due to their excellent multifaceted characteristics, such as high specific surface area, unique physicochemical properties, controllable electronic and mechanical properties, and tunable lateral size, novel two-dimensional nanomaterials are used in numerous applications and fields of research, such as biomedicine, energy storage, device fabrication and generation, and electronics [1–4].

**Fig. 1:**
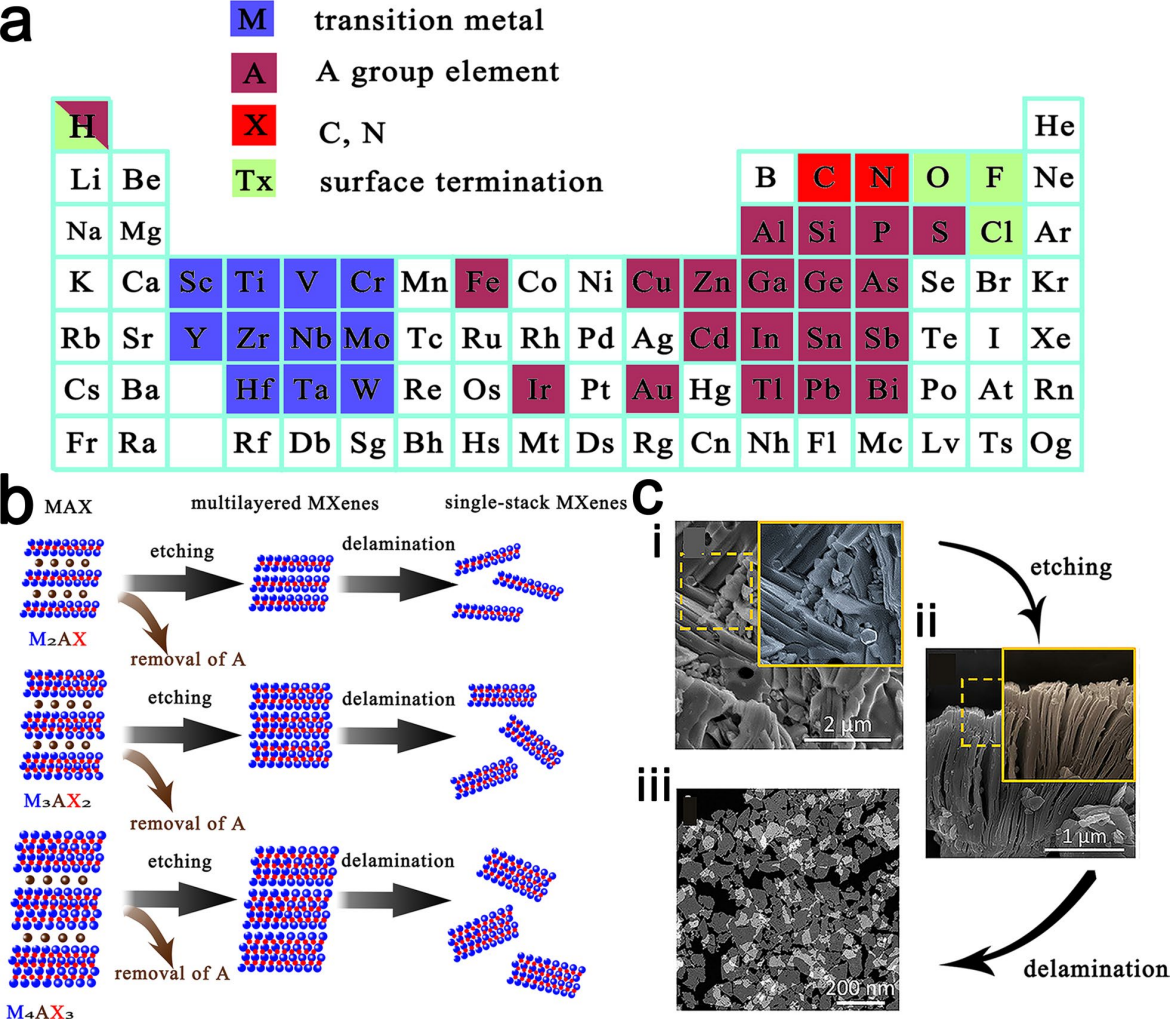
**a** Elements in the Periodic table that are known to form M_n+1_AX_n_ phases. **b** Structure of MAX phases and the corresponding MXenes. **c **(i) SEM images of Nb_2_AlC ceramic bulk (MAX phase). (ii) SEM image of multilayer Nb_2_CT_x_. (iii) Dark-field TEM image of single-layer Nb_2_CT_x_. Reproduced with permission from Ref. [18], © John Wiley and Sons 2017

MXenes are a new group of 2D inorganic materials with ultrathin atomic thicknesses that are composed of layered transition metal carbides and either nitrides or carbonitrides [5–7]. They share the simple structural formula M_n+1_X_n_ T_x_(n = 1–3), where M is an early transition metal (e.g., Ti, Nb, Cr, Ta, V, Sc, or Mo); X is carbon, nitrogen, or both [8–11]; and T_x_ represents the surface terminations (e.g., O, OH, F, and/or Cl) [12, 13], which form laminated structures with anisotropic properties (Fig. 1). The coordination ranges of these surface terminations can determine the surface properties of MXenes, of which the high coordination activities enable further surface functionalization of MXenes. MAX phases are layered hexagonal (space group P6_3_/mmc) stucture, where near-close-packed M-layers are interleaved with pure A-group element layers and X-atoms fill the octahedral sites between the M-layers [14]. In MAX phases, the M-X bond possesses both metallic properties and covalent bonding, whereas the M-A bond is metallic. Thus, the M-A bond is weaker than the M-X bond and more chemically active [15]. Therefore, the M_n+1_X_n_ structure can be obtained by selectively etching the A-layers from the precursor ternary-layered carbides of MAX phases, where A represents a group of 12–16 elements of the periodic table. Recently, another emerging family of 2D materials, 2D transition metal borides, has also attracted a great deal of attention and was named 'MBenes' in its early stages of discovery due to its perceived similarity to earlier MXenes [16, 17].

In 2011, Gogotsi et al. [14] produced multilayered Ti_3_C_2_T_x_ by etching Ti_3_AlC_2_ with hydrofluoric acid (HF) acid at room temperature, which was the first MXene. Since then, approximately 250 MXenes with various metal elements, C/N pairs, and surface terminations have been theoretically predicted in silico, while only approximately thirty species of MXenes have been successfully synthesized via experimental methods [19]. After the first observation, Rasoo et al. discovered in 2016 that 2D Ti_3_C_2_T_x_ can be used as antibacterial materials [20].

MXenes, which are novel 2D nanomaterials, have inherited many advantages of common 2D nanomaterials, such as extreme thinness, large specific surface area, high surface-to-volume ratio, and mechanical toughness. Additionally, abundant surface-terminating functional groups (e.g., hydroxyl (–OH), fluorine (–F), and oxygen (–O)) are present on their surfaces, which provide many active sites. This structure endows MXenes with hydrophilicity, provides abundant reactive sites for drug loading and enables flexible surface modification, functionalization, and scalable processability. Compared to conventional photosensitizers (PSs), MXene-based materials have strong absorption in both the first and second NIR biowindows and high light-to-heat conversion efficiency, thereby enabling their application in both photodynamic therapy (PDT) and photothermal therapy (PTT). Moreover, due to its excellent structural, biocompatible and electrical properties, MXene has attracted much attention in the development of biosensors. MXenes are highly desirable for application in various types of advanced biosensors, including fluorescent/optical, electrochemical and biocompatible field-effect transistor biosensors. And their properties are enhanced, by modifying the surface to augment MXenes characteristics or by combining them with other nanomaterials [21, 22]. Furthermore, studies have also demonstrated that several MXenes are biocompatible and nontoxic to living organisms, thereby opening new doors for applications in implants [23, 24]. In conclusion, with various surface modifications and functionalizations, MXene-based materials have great potential for various biomedical applications.

In this review, we aim to systematically summarize recent advances in MXene-based materials in biomedical applications. We review and discuss MXenes synthetic methods and surface chemistry, along with functionalization strategies. Afterward, we provide a detailed introduction to the biomedical applications of MXenes in various areas, including biosensors, diagnosis, implantation, antibacterial materials, and immunotherapy, among others. At the end of the review, we discuss the current challenges and future prospects of MXene-based materials in biomedical applications.

## Preparation of MXenes

Preparation methods for MXenes, which are a new family of 2D nanomaterials, have been extensively developed since the first discovery (Ti_3_C_2_T_x_) in 2011 by selective etching of the MAX precursor (Ti_3_AlC_2_) [14]. Generally, MXenes are obtained by eliminating the A-layers from their layered precursor MAX phases via selective etching. Recently, with further research on synthetic methods of MXenes, many methods have been developed, which can be divided into two main routes: a top-down approach that is based on direct exfoliation of multilayer bulk crystals and a bottom-up approach in which 2D ordered structures are grown from molecules/atoms. Moreover, to improve the properties of MXenes and endow them with new functionalities to meet the requirements of further biomedical applications, various surface modifications and functionalizations have been developed.

### Top-down approach

The top-down method is based on the direct exfoliation of bulk parent materials while retaining the original integrity. To remove van der Waals interactions between the stacked layers of the bulk parent materials, various driving forces are employed, including mechanical and chemical exfoliations.

#### Based on precursors

**Fig. 2:**
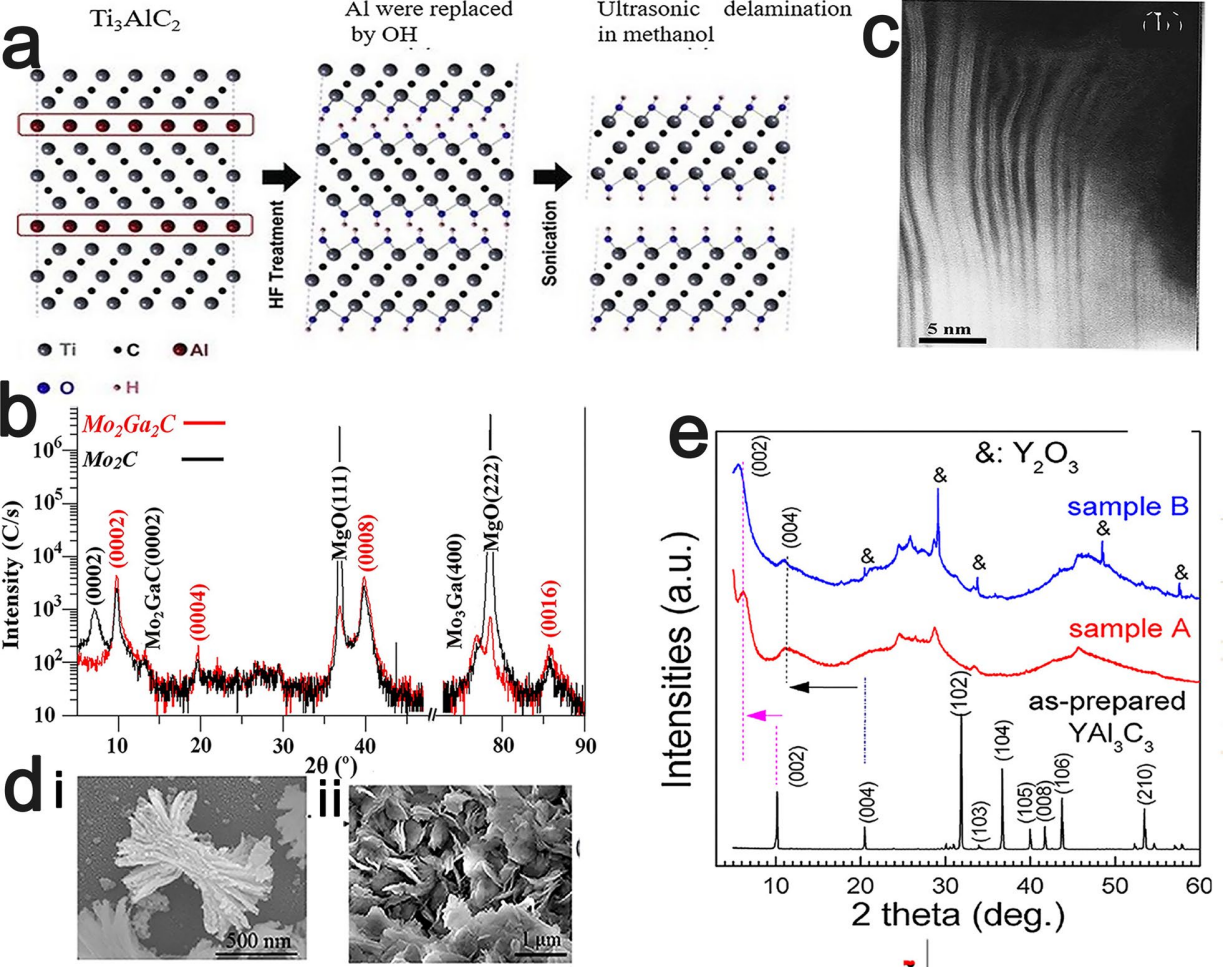
**a** Schematic diagram of the preparation process of Ti_3_AlC_2__._ Reproduced with permission from Ref. [14], © American Chemical Society 2017. **b** Symmetric 2θ X-ray diffractograms of Mo_2_Ga_2_C film before and after HF etching. Reproduced with permission from Ref. [25], © Elsevier 2015. **c** A higher magnification TEM micrograph of a Mo_2_Ga_2_C thin film after etching in 50% HF (aq). Reproduced with permission from Ref. [25], © Elsevier 2015. **d** SEM images of the 2D Zr_3_Al_3_C_5_ after HF treatment. Reproduced with permission from Ref. [26], © Elsevier 2020. **e** X-ray diffractograms of YAl_3_C_3_ before and after HF treatment. Reproduced with permission from Ref. [26], © Elsevier 2020

Based on the types of precursors, the preparation methods of MXenes can generally be divided into MAX-phase and non-MAX-phase methods. Eliminating the A-layers from the precursor MAX phases via selective etching is a typical method for producing MXenes. The typical preparation process is selective etching of the A-layer using an etchant at a suitable concentration for a specified time period, centrifugation or filtration to separate solid particles, and finally, sonication to obtain isolated sheets or monolayers. A typical example is the synthesis of the first Ti_3_C_2_T_x_ by Naguib et al. by selectively etching Ti_3_AlC_2_ with HF solution at room temperature (Fig. 2) [14].

In recent years, MXene synthesis from non-MAX phase precursors was also reported to produce MXenes. For instance, the synthesis of Mo_2_CT_x_, which was the first Mo-based MXene, from a non-MAX phase precursor by selectively etching Ga layers from Mo_2_Ga_2_C with a 50% concentrated HF solution was reported [25]. In contrast to any known MAX phase, two A-layers of Ga are stacked between the Mo_2_C layers. Comparing the X-ray diffraction patterns of Mo_2_Ga_2_C and Mo_2_CT_x_ before and after the etching treatment (Fig. 2b), there is a significant reduction in the peak intensity of the impurity phase (Mo_3_Ga) from Mo_2_Ga_2_C, which indicates that this phase was largely dissolved during the etching process. A TEM image that was captured after HF etching (Fig. 2c) shows clear delamination, thereby indicating that the A-layer was removed. YC_x_ that is derived from the non-MAX phase precursor YAl_3_C_3_ is another example, where the Al-C layers need to be etched instead of pure Al layers in the typical method [26]. An SEM image of YAl_3_C_3_ that was captured after HF treatment shows an obvious grain with an accordion morphology (Fig. 2d), which is similar to the morphology of the previous MXene that was synthesized based on the MAX phase. Comparing the X-ray diffraction patterns of YAl_3_C_3_ before and after HF treatment (Fig. 2e), the significant reduction of the corresponding Al signal after etching indicates the removal of the Al layer from the original crystal structure.

#### Based on delamination intercalants

In general, the synthesized MXenes are multilayered and require further processing to obtain monolayer MXenes. Currently, the methods of delamination of multilayer MXenes are divided into two types: mechanical exfoliation and delamination by intercalation. The interaction between the multilayered MXenes that is produced by etching is so sticky (the interlayer interaction is approximately 2–sixfold stronger than that of graphite or bulk MoS_2_ [27]) that it is not efficient enough to produce single-stack MXenes by simple mechanical exfoliation [28]. In addition, prolonged sonication may have a negative effect on lamellar structures, decrease the size of MXene sheets and even increase the defect rate of MXenes [29, 30]. Therefore, the use of intercalants is suggested to decrease the interlayer spacing and weaken the interactions between MXene layers, thereby subsequently increasing the yield of delamination [29, 31]. The intercalators that are widely used for the intercalation of MXenes can be commonly classified into two main types: organic intercalants and ionic aqueous solution intercalants. The organic intercalants include polar organic molecules (e.g., DMSO [32], isopropylamine [32, 33], urea, and hydrazine) and large organic base molecules (e.g., TBAOH [34, 35], TPAOH [36], n-butyllithium [37], and choline hydroxide). The ionic aqueous solution intercalants include metal hydroxides or halide salts in aqueous solutions, which are suitable for the delamination of large MXenes [38].

#### Based on etchants

In terms of etchant composition, we can classify the preparation methods into HF-etching and non-HF-etching approaches.

##### HF etching

The MXene synthesis methods of the first type involve the application of aqueous HF acid at a suitable concentration within a specified time period, which are typically used to produce multilayered MXenes that are stabilized through hydrogen bonding and van der Waals interactions. In this process, layered MAX phase powders are stirred with aqueous HF acid at room temperature. As a result, the A-layers of the MAX phase can be selectively and easily removed, and M–A bonds are replaced by weak interactions of T_x_ terminations such as hydroxyl (–OH), fluorine (–F), and oxygen on the surfaces of multilayered MXenes. Then, multilayered MXenes are easily delaminated and intercalated through delamination intercalant intercalators or sonication treatment to fabricate single-layer MXenes.

**Fig. 3:**
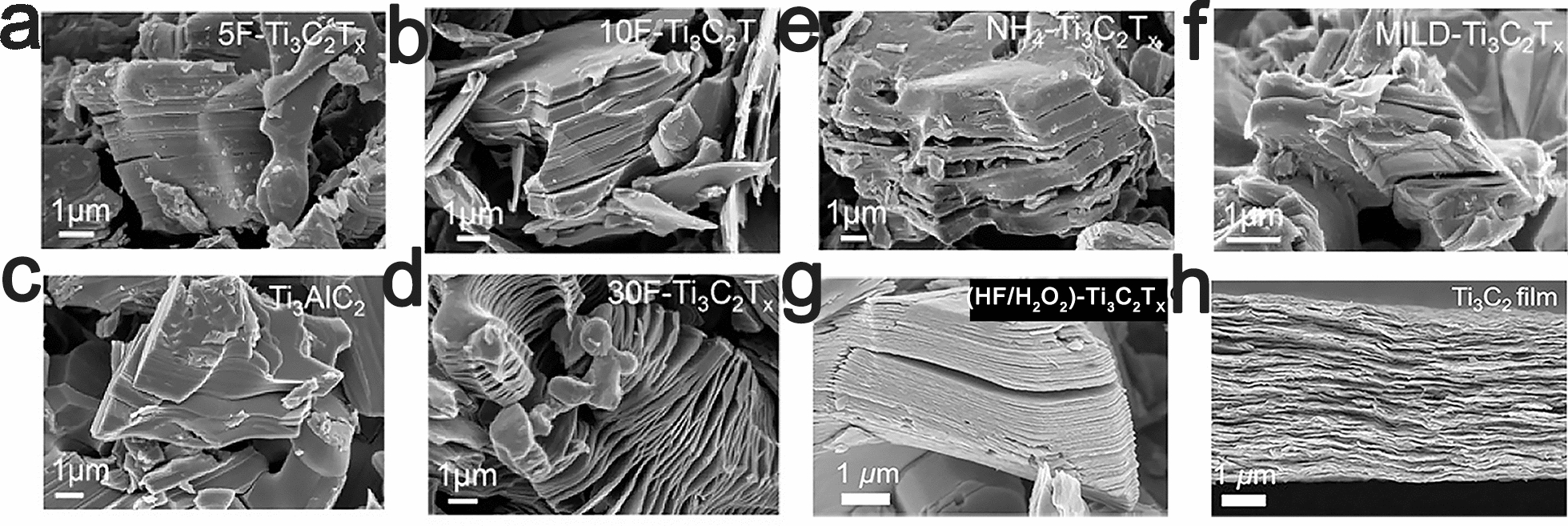
SEM images of MAX and MXene powders. SEM images of multilayer Ti_3_C_2_Tx powders synthesized by etching with 30 wt% (**a**), 10 wt% (**b**) and 5 wt% HF (**c**) [39]. **d** SEM images of Ti_3_AlC_2_(MAX) powder [39]. SEM images of Ti_3_C_2_Tx powders synthesized with ammonium hydrogen fluoride (**e**) and 10 M LiF in 9 M HCl (**f**). Reproduced with permission from Ref. [39], © American Chemical Society 2017. **g** SEM images of Ti_3_C_2_T_x_generated by (HF/H_2_O_2_)-treated Ti_3_SiC_2_ [44]. **h** A cross-sectional SEM image of Ti_3_C_2_T_x_ films made by vacuum-assisted filtration of a colloidal solution of Ti_3_C_2_T_x_ in TMAOH. Reproduced with permission from Ref. [44], © John Wiley and Sons 2018

According to several studies in recent years, various etching parameters, such as temperature, etching time and HF etchant concentration, play a determinant role in the quality of the prepared MXene nanosheets [39–41]. Gogotsi et al. demonstrated that an excessively high concentration of HF etchant generates more defects in Ti_3_C_2_T_x_ MXenes but an excessively low concentration cannot form accordion-like structures (Fig. 3) [39, 40]. M_n+1_X_n_T_x_ recrystallizes or decomposes at high temperatures [41]. To obtain favorable nanosheets, various types of M_n+1_AlX_n_ require different etching parameters [39, 42]. Moreover, different wet techniques can create different surface properties, thereby resulting in the generation of MXenes with various characteristics.

Since Ti_3_SiC_2_ has remarkable tolerance to bases and strong acids (including HF), Ti_3_C_2_T_x_ cannot be produced by etching Ti_3_SiC_2_ in HF or other etchants [43]. Gogotsi et al. recently developed a new method for the large-scale synthesis of Ti_3_C_2_ via oxidant-assisted selective etching of silicon from Ti_3_SiC_2_, which greatly widens the range of precursors for MXene synthesis [44]. It was shown that various HF oxidants (e.g., HF/H_2_O_2_, HF/(NH_4_)_2_S_2_O_8_, HF/KMnO_4_, HF/FeCl_3_, and HF/HNO_3_) can be employed to etch Si layers from Ti_3_SiC_2_ to produce Ti_3_C_2_T_x_ (Fig. 3), which has the same structure as Ti_3_C_2_T_x_ that was derived from Ti_3_AlC_2__,_ as confirmed by microscopy.

##### Non-HF-etching

Although HF is widely and effectively utilized for MXene synthesis, it is highly corrosive and harmful to humans and the environment [15]. A trace amount of remaining HF during the etching process could induce cell death, thereby harming the health of biological organisms. Therefore, studies have increasingly started to explore the preparation of MXenes by non-HF etching methods.

One approach is to use the HF that is formed in situ through the reaction of acids with fluorides (usually HCl/LiF and HCl/NaF) to selectively etch the A-layer. Although the properties of MXenes that are synthesized by this method are similar to those of MXenes that are prepared using HF, the synthesis process is less hazardous, and the in situ HF etching method avoids the toxicity of MXenes during the synthesis process. Ghidiu et al. used a mixture solution of HCl and LiF in which Ti_3_AlC_2_ powders were selectively etched at 40 °C for 45 h to prepare Ti_3_C_2_T_x_, which was a safer and faster route for fabricating MXenes at high yield [45]. As observed by transmission electron microscopy (TEM) of Ti_3_C_2_T_x_, the synthesized Ti_3_C_2_T_x_ had fewer defects.

MXenes can also be obtained by another safe and effective method with weakly acidic and environmentally friendly bifluoride etchants, such as KHF_2_, NaHF_2_ and NH_4_HF_2_. Yu et al. successfully synthesized Ti_3_C_2_T_x_ MXenes with large interplanar spacing by etching Ti_3_AlC_2_ with bifluoride (NaHF_2_, KHF_2_, and NH_4_HF_2_) in a single-stage process [46]. During the etching process, NH^4+^ Na^+^, or K^+^ ions enter the interlayer space of MXenes, further enlarging the interplanar spacing and promoting the delamination efficiency. Natu et al. also experimentally demonstrated that Ti_3_C_2_T_x_ flakes rich in fluorine terminations can be obtained by etching with NH_4_HF_2_ in an organic polar solvent [47].

Compared to M_n+1_AlC_n_, Al atoms are more strongly bonded in M_n+1_AlN_n_; thus, more energy is required for their removal. M_n+1_AlN_n_ is less stable and might dissolve in HF solutions [48]. For these reasons, it is difficult to eliminate the A-layer from nitride-based MAX phases. The third approach is to use molten fluorides with the assistance of high-temperature heating. Under these conditions, the free F^−^ is active enough to etch the A-layer from the MAX phase. In 2016, Urbankowski et al. heated Ti_4_AlN_3_ in a eutectic fluoride salt mixture (59 wt% KF, 29 wt% LiF and 12 wt% NaF) under an argon (Ar) atmosphere at 550 °C for 30 min and obtained the first nitride-based MXene (Ti_4_N_3_T_x_) [49]. In 2020, Kamysbayev et al. synthesized bromide-terminated MXenes by etching the MAX phase in molten CdBr_2_. In contrast to the strong Ti-F and Ti–O bonds that are produced by etching with conventional methods (e.g., HF), which have difficulty performing post-synthesis covalent surface modifications of MXenes, they demonstrated that the labile surface bonds of Cl- and Br-terminated MXenes render them more readily available as versatile synthons for further chemical transformations [8].

Studies [28, 50, 51] have shown that the etchant is an important factor in determining the surface termination of MXenes. For example, fluorine-containing etchants can increase the abundance of F on the surface of MXenes, thereby leading to decreases in the abundances of other functional groups, such as oxy groups and hydroxyl groups. Thus, MXenes that are prepared using fluorine-containing etchants usually require further modification before biomedical application. To facilitate the preparation of MXenes for biomedical applications, etching with fluorine-free etchants can be performed to produce controllable functional surface termination of MXenes. Recently, many HF-free methods have been applied to produce MXenes. In 2017, fluorine-free synthesis of MXenes was reported by Urbankowski et al. [50]. In this study, V_2_NT_x_ and Mo_2_NT_x_ were converted from V_2_CT_x_ and Mo_2_CT_x_ via ammonification at 600 °C, in which the C atoms were replaced by N atoms. The produced Mo_2_N retained the structure of the MXenes, while V_2_CT_x_ transformed into a mixed layered structure of cubic VN and trigonal V_2_NT_x_. In 2018, Li et al. reported the synthesis of Ti_3_C_2_T_*x*_ by 27.5 M NaOH at 270 °C, thereby providing an alkali‐etching strategy for synthesizing new MXenes. In 2019, Hao et al. synthesized Ti_2_CT_x_ by E-etching with a composite electrode, which provided a universal strategy for synthesizing MXenes based on a thermal-assisted electrochemical etching route [51].

### MQDs

Compared to 2D MXenes, MXene-derived quantum dots (MQDs) exhibit superior properties, including easier functionalization, more attractive electronic and magnetic properties, and excellent photoluminescence (PL) properties because of their higher surface area (namely, lateral size of usually < 10 nm) and forceful quantum confinement effect while inheriting the advantages of 2D MXenes [52]. Currently, hydrothermal methods are considered to be the most common method for the preparation of MQDs. Apart from hydrothermal/solvothermal methods, other synthesis methods for MQDs have also been extensively developed recently, including microwave-assisted synthesis [53], reflux [54], ultrasonic [55, 56], intercalation and combined methods [57]. In 2017, Huang et al. reported the preparation of the first Ti_3_C_2_ MQDs by a facile hydrothermal method, which was of great significance for broadening the application areas of MXenes [58]. In a recent report, an in situ strategy employing a temporally and spatially shaped femtosecond laser (TSBL) is used to photochemically synthesize MQDs [59]. The temporal shaping of the unique TSBL enables effective control of the multilevel photoexfoliation of MXenes and water photoionization–enhanced light absorption for creating MQDs. Of course, in addition to top-down methods using bulk materials as precursors, MQDs can also be synthesized from small organic and inorganic molecular precursors via bottom-up methods [60].

### Bottom-up approach

MXenes can also be synthesized through crystal growth using small organic and inorganic molecules as precursors. Instead of top-down approaches, which lack size distribution controllability and reproducibility, bottom-up synthesis approaches have the advantages of enabling precise manipulation of the size distribution, geometric morphology and surface termination of MXenes [61–64]. Meanwhile, due to their relatively simple implementation, bottom-up approaches are expected to greatly improve the yield of MXenes in the future. However, compared to top-down fabrication methods, few studies have been conducted on bottom-up synthesis methods of MXenes, which is perhaps due to the complex structures of MXenes and multicomponent atom layers.

In 2015, Ren et al. reported the fabrication of high-quality, excellent-stability and defect-free ultrathin α-Mo_2_CT_x_ crystals by chemical vapor deposition (CVD) [61]. They synthesized 2D ultrathin α-Mo_2_CT_x_ crystals with lateral sizes of over 100 μm on a Cu/Mo foil under a methane atmosphere at temperatures above 1085 °C, where methane acted as the carbon source. Recently, non-laminated stacked Mo_2_N sheets have also be achieved on Cu/Mo substrates by using CVD with NH_3_ as the nitrogen source under the temperature of 1080 °C [62]. In 2020, Turker et al. performed a detailed study on the effects of reaction temperature, reaction time, copper layer thickness and other factors during the CVD reaction, and finally, they found that the Mo_2_C crystals that formed on the graphene surface were thinner and had fewer defects than those that formed on the copper surface [63].

In addition to CVD, pulsed laser deposition (PLD) and salt template methods have also been developed for the preparation of MXenes. The face centered cubic structured Mo_2_C thin film were obtained by PLD using a methane plasma as the carbon source and a pulsed laser to ablate a Mo metal target, which was heated to a growth temperature of 700 °C on a sapphire substrate [64]. Firstly, 2D hexagonal MoO_3_-coated NaCl (2D h-MoO_3_@NaCl) was obtained by annealing the Mo precursor@NaCl powders in Ar atmosphere at 280 °C. Then, the 2D h-MoO_3_@NaCl powder was slowly ammoniated in a NH_3_ atmosphere at 650 °C to prepare 2D MoN@NaCl powders. Finally, the 2D MoN@NaCl powders were washed in deionised water and further filtered to remove the salts [65].

Therefore, although the current research on bottom-up synthesis methods for A is relatively difficult, it is promising, and more attention should be given to these strategies. A summary of MXenes synthesis methods is shown in Table 1.

**Table 1:** Summary of MXenes synthesis methods

Synthesis method			MAX-phase	MXene	Etchant	Temperature	Delamination	Features	Refs.
Type of precursors	MAX-phase	Metal	Ti_3_AlC_2_	Ti_3_C_2_	HF	RT	Without delamination	Traditional synthesis, which has been extensively studied	[14]
	No-MAX-phase		YAl_3_C_3_	YC_x_	HCL/LiF HF		DMSO	Emerging synthetic methods for specific metal elements such as the transition metals Zr and Hf	[26]
			Hf_3_[Al(Si)]_4_C_6_	Hf_3_C_2_	HF		Without delamination		[66]
			Mo_2_Ga_2_C	Mo_2_C	HF	50 °C	Without delamination		[25]
Based on etchants	HF-etching	HF	Ti_3_AlC_2_ Nb_2_AlC	Ti_3_C_2_ Nb_2_C	HF HF	RT RT	Without delamination TPAOH	Disadvantages: It is highly corrosive and harmful to humans and the environment. Remaining HF could induce cell death, thereby harming the health of biological organisms	[67] [18]
		HF/Oxidizing agent	Ti_3_SiC_2_	Ti_3_C_2_	HF/H_2_O_2_	40 °C			[44]
	Non-HF-etching	Situ HF etching	Ti_3_AlC_2_	Ti_3_C_2_	HCl/LiF	40 °C	in distilled water via ultrasonication	Advantages: This method avoids the toxicity of MXenes during the synthesis process and is less hazardous	[45]
		Bifluoride solutions	Ti_3_AlC_2_	Ti_3_C_2_	KHF_2_ or NaHF_2_	60 °C	Without delamination	Advantages: During the etching process, NH^4+^ Na^+^, or K^+^ ions could enter the interlayer space of MXenes, further enlarging the interplanar spcccscacing and promoting the delamination efficiency	[46]
			Ti_3_AlC_2_	Ti_3_C_2_	NH_4_HF_2_	60 °C	dimethyl sulfoxide via ultrasonication under argon		[68]
		Molten fluorides	Ti_4_AlN_3_	Ti_4_N_3_	LiF, NaF, KF	550 °C	TBOAH and Sonication	Advantages: Compared to M_n+1_AlC_n_, Al atoms are more strongly bonded in M_n+1_AlN_n_, so it is difficult to eliminate the A-layer from nitride-based MAX phases. This approach using molten fluorides with the assistance of high-temperature heating can provide higher energy to etch the A-layer from the MAX phase	[49]
			Ti_3_AlC_2_	Ti_3_C_2_	CdBr_2_		N-methyl formamide		[8]
		Fluorine-free	Ti_3_AlC_2_	Ti_3_C_2_	NH_4_OH	RT	Without delamination	Advantages: Etching with fluorine-free etchants can be performed to produce controllable functional surface termination of MXenes	[67]
			Ti_2_AlC	Ti_2_CT_x_	electrochemical etching				[51]
			V_2_C Mo_2_C	V_2_N Mo_2_N	ammonification	600 °C			[50]
			Ti_3_AlC_2_	Ti_3_C_2_T_*x*_	NaOH,	270 °C	DMSO		[69]
Bottom-up approach		CVD		Mo_2_N				Advantages: Enabling precise manipulation of the size distribution, geometric morphology, surface termination of MXenes and simple implementation	[62]
				Mo_2_C					[63]
		PLD		Mo_2_C					[64]
		Salt template methods		W_2_N, V_2_N					[65]

### Surface modification and functionalization

**Fig. 4:**
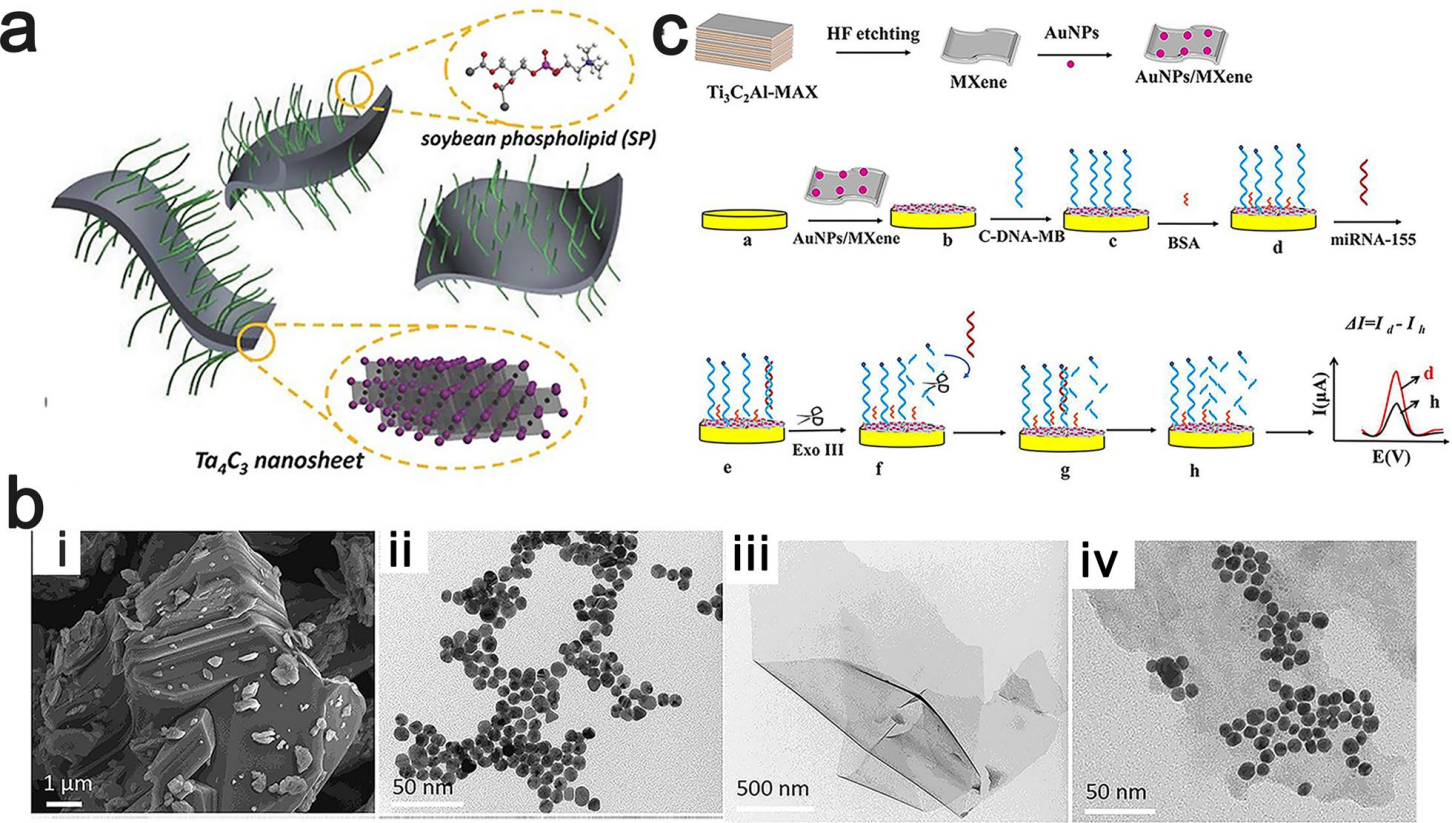
**a** Schematic diagram of the surface modification of Ta_4_C_3_ nanosheets using SP. Reproduced with permission from Ref. [71], © John Wiley and Sons 2017. **b** (i)The SEM image of Ti_3_AlC_2_. (ii)TEM images of AuNPs. (iii) TEM images of Ti_3_C_2_T_x_ nanosheets. (iv) TEM images of AuNPs/Ti_3_C_2_ nanocomposites. Reproduced with permission from Ref. [73], © Elsevier 2020. **c** Schematic diagram of the preparation of electrochemical miRNA-155 biosensor based on AuNPs/Ti_3_C_2_ 3D nanocomposite. Reproduced with permission from Ref. [73], © Elsevier 2020

The large surface area and abundant functional groups of MXenes provide the basis for the surface modification of MXenes. Although the excellent physicochemical properties of MXenes have given MXenes the potential to be used in several fields of application, even with more in-depth research on MXene-based materials, these properties still cannot meet the requirements of various applications. There are still some defects of MXenes in vivo, including poor water dispersibility, slow degradation and toxicity [70]. Therefore, surface modification and functionalization are needed to enhance the properties of MXene-based materials and impart new functions to these materials. At present, research on the surface modification of MXenes is focused on two strategies: the first is a polymer-based surface chemistry strategy, which is based on noncovalent or covalent interactions to immobilize selected molecules or polymers on the MXene surface. It was shown that the synthesized MXene nanosheets were less stable in biological media and were prone to aggregation and precipitation. To improve the stability of MXene nanosheets in physiological environments, researchers modified the surfaces of Ta_4_C_3_T_x_ nanosheets with soybean phospholipids (SPs) to reduce the zeta potential of the Ta_4_C_3_T_x_ nanosheets (Fig. 4a), which effectively improved the colloidal stability of the Ta_4_C_3_ nanosheets in physiological environments [71]. In addition, the modification of Ti_3_C_2_T_x_ by aryl diazo grafting with derivatives that contain sulfonyl or carboxyl betaine side groups has promoted the development of MXene-based functional enzymes and affinity-based electrochemical biosensors [72].

The other strategy is based on the surface chemistry of inorganic nanoparticles, which combines inorganic nanomaterials with MXenes with multifunctionalities to further broaden their potential applications. For instance, AuNPs/Ti_3_C_2_T_x_ nanocomposite prepared by immobilizing Au nanoparticles on the surfaces of Ti_3_C_2_T_x_ (Fig. 4b) had the combined advantages of a large specific surface area and excellent electrical conductivity, and was successfully used to develop an ultrasensitive electrochemical biosensor for the detection of miRNA-155 (Fig. 4c) [73]. Similarly, combining Au, Fe_3_O_4_ and MXenes, the prepared Au/Fe_3_O_4_/MXene composites showed less toxicity than the pure MXenes in in vitro and in vivo experiments, thereby promoting the application of MXenes in Photothermal therapy [74].

In addition, in a recent study, Wan et al. made full use of the abundant surface functional groups on MXene surfaces and used sequential bridging of hydrogen and covalent bonding agents to achieve effective densification and removal of voids in MXene films, thereby leading to highly compact MXene films. The mechanical strength and toughness, electrical conductivity and electromagnetic interference shielding ability of the obtained MXene films have been greatly improved [12].

In conclusion, the rapid development of MXene synthesis methods will also promote more and broader applications of MXenes in the biomedical field.

## Biomedical applications

Due to the fascinating physicochemical properties of MXenes, MXenes and their composites have been developed for various biomedical applications, including biosensing, bioimaging, therapeutic diagnostics, implants and antibacterial agents.

### Biosensors

In comparison with conventional metal nanoparticle-based biosensors, new nanomaterials, including carbon nanotubes and graphene, have shown various advantages in biosensing. As novel nanomaterials, MXenes have attracted much attention in biosensor development due to their outstanding structural properties, excellent biocompatibility and superior electrical properties. As high-performance receptors, they have high selectivity, a low limit of detection (LOD), high sensitivity, a short response time, and a wide linear range, which are the main performance parameters. Of course, they should also have a low production cost to facilitate commercial scale-up production. The applications of MXenes in biosensors are divided into three main categories: electrochemical biosensors, fluorescent/optical biosensors and biocompatible field-effect transistors. The MXenes applications in biosensors is summarized in Table 2.

**Table 2:** Summary of MXene applications in biosensors

Type			Material	Target	Detection limit	Linear range	Sensitivity	Stability	Refs.
Electrochemical biosensors	Enzyme-based biosensors		Ti_3_C_2_T_x_ MXene/β-HBD	β-hydroxybutyrate	44.5 μM	360 μM–17.91 mM	0.480 μA·mM^−1^·cm^−2^	Retained approximately 97.08% of its initial response to β-HBA after 7 days and 93.20% after 30 days	[77]
			Tyr-Ti_3_C_2_T_x_ MXene	Phenol	12 nM	0.05–15.5 μM	414.4 mA·M^−1^	Deviation (RSD) was 1.6%, for 7 successive determinations,	[78]
			Ti_3_C_2_T_x_MXene/graphene (MG)	Glucose			Detection sensitivity: 12.10 µA·mM^−1^	Negligible current decrease over 300 scanning cycles	[80]
	Affinity Sensors	Nucleic acid-based biosensors	Ti_3_C_2_T_x_MXene	Gliotoxin	5 pM	5 ~ 10 nm			[83]
			Au/Ti_3_C_2_T_x_MXene	MicroRNA	microRNA-21: 204 aM microRNA-141: 138 aM	500 aM ~ 50 nM			[84]
		Immunosensors	Ti_3_C_2_-MXene/GC	Carcinoembryonic antigen, CEA		0.1 pg–2 mg/mL^−1^	37.9 µA·ng^−1^·mL·cm^−2^		[88]
		Molecular imprinting sensors	Ti_3_C_2_T_x_ MXene	Amyloid-β protein	0.3 fg·mL^−1^	1.0 fg·mL^−1^ ~ 100.0 fg·mL^−1^			[87]
Fluorescent/Optical biosensors		Fluorescent quenching agent	a Cy3-labeled CD63 aptamer (Cy3-CD63 aptamer)/Ti_3_C_2_T_x_ MXenes	Exosome	1.4 × 10^3^ mL^–1^	10^4^ ~ 10^9^ mL^–1^			[92]
			FAM-labeled ssDNA probe/Ti_3_C_2_T_x_ MXene	HPV-18	100 pm				[97]
		MQDs	Ti_3_C_2_ MQDs	Fe^3+^	310 nM	5 ~ 1000 μM		The RSD of the sensor for 10 and 250 μM of Fe^3+^ was 1.1% and 1.2%,	[56]
			ε-poly-L-lysine (PLL)/Ti_3_C_2_ MQDs	Forcyt-c, trypsin	cyt-c: 20.5 nM; trypsin: 0.5 ~ 80 μg/mL	cyt-c: 0.2 ~ 40 μM; trypsin: 0.1 μg/mL			[93]
FET			Ti_3_C_2_T_x_MXene	Dopamine	100 nM	100 nM ~ 50 mM			[94]

#### Electrochemical biosensors

Electrochemical biosensors rely on biological recognition elements and offer lower detection limits and high sensitivity and selectivity toward target analytes. The basic principle of biosensors of this kind is that the recognition between immobilized biomolecules and target analytes leads to changes in the electrical properties of the sensing material or solution, such as its conductance, potential, electric current, and ionic strength, which can be detected by amperometry, potentiometry, voltammetry, and impedance techniques [75, 76].

To date, MXenes that are composed of various elements have been successfully synthesized, but only the application of Ti_3_C_2_ has been reported for electrochemical biosensing.

##### Redox proteins/enzyme-based electrochemical biosensors

Enzymes, which were the first biological recognition elements to be applied in the field of biosensors, can effectively and selectively react with target analytes, thereby ultimately triggering an electrochemical response. By immobilizing enzymes on MXenes to develop biosensors, the enzymes can provide selectivity to the biosensors, while the MXenes act as transducers to take full advantage of their electrical conductivity, large surface area and high biocompatibility.

**Fig. 5:**
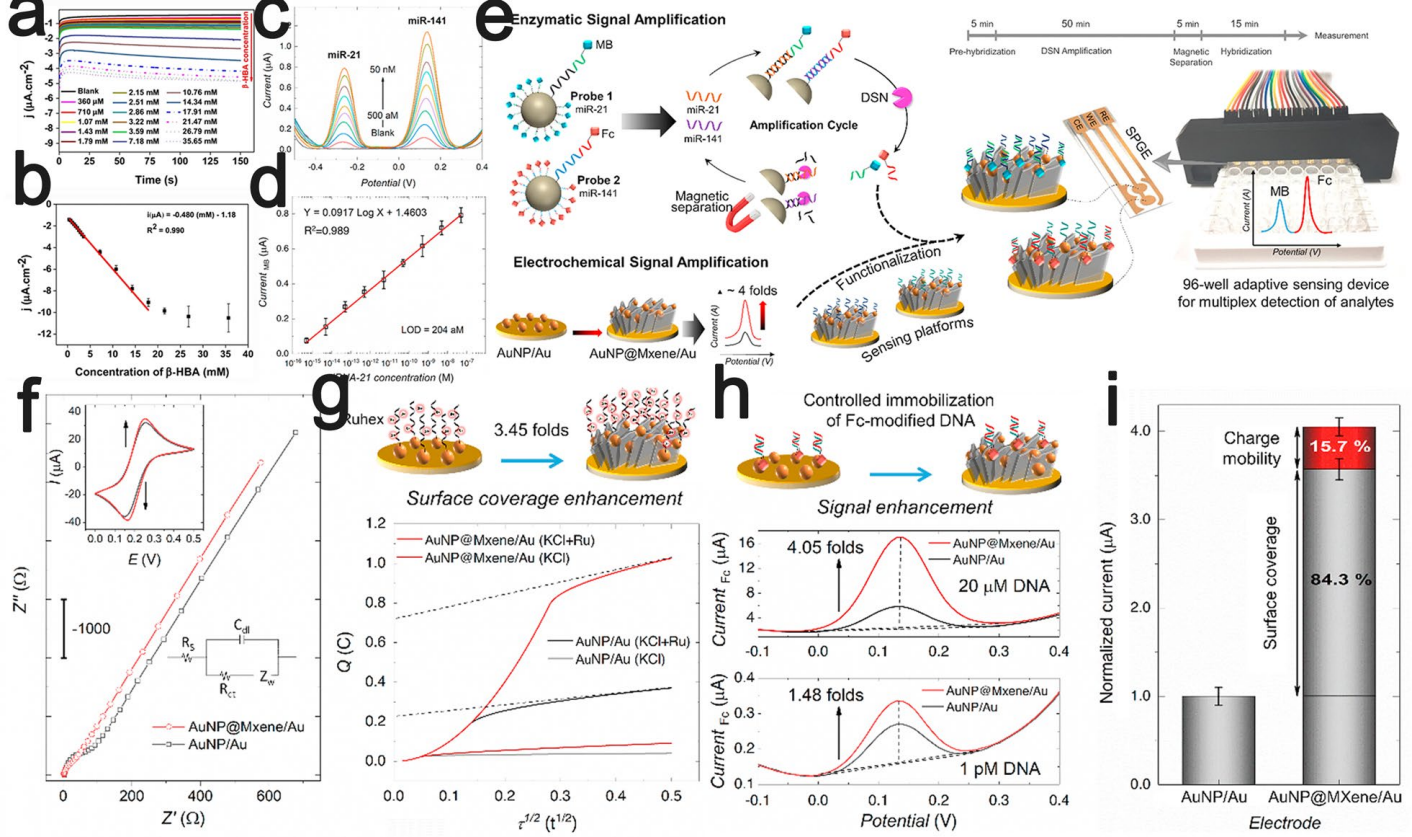
**a** The amperometry *i*-*t* curve obtained at the Au-PCB/Ru/MXene-β-HBD-NAD-GA-BSA electrode at an applied potential of −0.35 V vs. Ag/AgCl (3 M NaCl) in PBS pH 7.4 **b** The corresponding linear calibration plot for the amperometric determination of β-HBA (*n* = 3)^+^. Reproduced with permission from Ref. [77], © Springer Nature 2020. **c** Typical DPV response of the fabricated biosensor device toward multiple detection of serial concentrations of miR-21 and miR-141 in HEPES buffer (pH 7.4). **d** Corresponding regression plot illustrating the oxidation current peak values of MB and Fc as a function of miR-21 concentrations. **e** Schematic diagram representing the whole assay procedure for multiplex and concurrent detection of miR-21 and miR-141. **f** Nyquist plots (Z′ vs.—Z″) obtained for AuNP/Au and AuNP@MXene/Au and the equivalent Randles circuit model; Inset shows the corresponding cyclic voltammograms of AuNP/Au (black) and AuNP@MXene/Au (red); Experiments were performed in PBS (pH 7.4) comprising 5 mM of Fe (CN)_6_^4−/3−^ and 0.1 M KCl with CV scan rate of 50 mV/s. **g** Typical chronocoulometric response of RuHex on AuNP/Au and AuNP@MXene/Au in 20 mM KCL and hexaammineruthenium (iii) chloride (200 μM) + KCl (20 mM); Dash lines show the outward stretching tangents extrapolated to the Y-axis illustrating the intercept values. **h** DPV curves obtained for Base^141^/AuNP/Au and Base^141^/AuNP@MXene/Au after being hybridized with uncleaved Fc-labeled DNA sequences (DSN products of 20 μM and 1 pM miR-141 reaction). **i** Statistical analysis of the normalized oxidation current peak value of Fc. Reproduced with permission from Ref. [84], © Elsevier 2020.

The large surface area of MXenes with unique laminar morphology can provide large-area immobilization of enzymes. Lee et al. prepared enzymatic beta-hydroxybutyrate biosensors for amperometric sensing using β-hydroxybutyrate dehydrogenase-modified Ti_3_C_2_T_x_-type MXene nanosheets, which were successfully applied to the determination of β-hydroxybutyrate in (spiked) real serum samples [77]. The laminar deconstruction of Ti_3_C_2_T_x_ facilitated the immobilization and encapsulation of the enzyme, thereby providing a suitable microenvironment for β-hydroxybutyrate dehydrogenase (β-HBD) to retain bioactivity and stability for a long time. The prepared biosensors displayed a low detection limit of 44.5 μM, a sensitivity of 0.480 μA·mM^−1^·cm^−2^ and a wide linear range of 360 μM—17.91 mM for β-HBA (Fig. 5a and b).

The surfaces of MXenes have abundant functional groups. Most of the MXenes that have been developed for electrochemical biosensors are prepared by etching with aqueous hydrofluoric solution, thereby generating more hydroxyl surface terminations. Therefore, the MXene surfaces are hydrophilic, which facilitates uniform dispersion in aqueous media and provides a good microenvironment for enzyme immobilization. The MXene-based tyrosinase biosensors that were fabricated by Wu et al. exhibited good analytical performance in the linear range of 0.05–15.5 μmol/L with a sensitivity of 414.4 mA·M^−1^ and a low detection limit of 12 n mol/L [78]. In this study, Ti_3_C_2_T_x_ nanosheets were synthesized by exfoliating the Al layer from the precursor Ti_3_AlC_2_ with HF at room temperature; consequently, the surface of Ti_3_C_2_ was terminated mostly by -OH, which provided an aqueous-like biocompatible microenvironment for immobilized enzyme molecules.

Although MXene-based biosensors alone have exhibited satisfactory results, constructing MXene composites is an excellent strategy. The construction of composite materials enables the various materials to complement each other to produce better results, such as overcoming electrode surface resistance upon enzyme immobilization [79] and enhancing the affinity and stability of graphene for enzymes [80]. TiO_2_ has superior biocompatibility and chemical stability. To further improve the biocompatibility of Ti_3_C_2_T_x_ (MXene), Wang et al. modified Ti_3_C_2_T_x_ with TiO_2_ to maintain protein bioactivity and stability more effectively [81]. They synthesized TiO_2_ nanoparticles that were modified with Ti_3_C_2_ nanocomposites by simple in situ hydrolysis followed by a hydrothermal process. Nanoscale TiO_2_ with a size of less than 30 nm greatly increased the effective surface area for protein adsorption. Ti_3_C_2_T_x_, which has an excellent charge transfer rate, is an efficient energy transfer medium between the enzyme and the electrode. Hence, using hemoglobin (Hb) as a model protein, the constructed TiO_2_–Ti_3_C_2_-based biosensor displayed good performance for the detection of H_2_O_2_ with a low detection limit, a wide linear range and especially excellent long-term stability, thereby offering a new avenue for broadening the applications of MXenes in enzyme immobilization. As another example, Rakhi et al. successfully demonstrated a GO_x_/Au/MXene/Nafion/GCE biosensor by a dropcasting method in which anchored Au nanoparticles significantly improved the electron transfer process between GO_x_ and GCE [79]. As a result, the GO_x_/Au/MXene/Nafion/GCE biosensor was shown to be suitable for the detection of glucose concentrate (in a range 0.1–18 mM) in biological samples with good sensitivity of 4.2 μA·Mm^−1^·cm^−2^, a lower detection limit of 5.9 μM, and excellent stability, reproducibility and repeatability. Moreover, many other methods are available for developing novel biosensors by the hybridization of MXenes with other suitable nanomaterials.

##### Affinity sensors

Affinity sensors specifically recognize analytes and form stable complexes with them. According to the type of analyte binding on the affinity sensor, affinity sensors can be classified into nucleic acid-based biosensors, molecularly imprinted polymer (MIP) sensors and immunosensors.

Nucleic acid sensors are based on nucleic acids, which are used as molecular recognition elements and are immobilized on the surface of an MXene-based material with a large specific surface area. Nucleic acid-based biosensors are also known as genosensors or aptasensors and can be further divided into RNA sensors and DNA sensors [82].

An example is tetrahedral DNA nanostructures (TDNs), which have a unique configuration that enables efficient and rapid binding of target molecules onto the electrode surface, thereby producing amplified electrochemical signals. In 2019, Wang et al. immobilized tetrahedral DNA nanostructures onto the surfaces of Ti_3_C_2_T_x_ nanosheets through the coordination interactions of phosphate groups on DNA with titanium to prepare a highly sensitive electrochemical biosensor for the detection of gliotoxin [83]. Combining the advantages of the large surface area of Ti_3_C_2_T_x_ and the molecular recognition of TDNs, the prepared sensor exhibited a wide detection range from 5 pM to 10 nM with a low limit of detection (LOD) of 5 pM. Moreover, a synergetic MXene-based and duplex-specific nuclease (DSN)-based signal amplification system was also reported [84], which was applied on an SPGE electrode for the very sensitive, specific and rapid detection of multiple miRNAs in total plasma,. MXene-Ti_3_C_2_T_x_ was modified with 5 nm AuNPs, which significantly enhanced the electrochemical properties of the MXene (Fig. 5c–i). Of course, the use of RNA as a receptor in the design of biorecognition elements for label-free ultrasensitive detection of miRNA-182 has also been reported [85].

Molecularly imprinted polymers, which are modified materials that improve the selectivity of sensors, have the advantages of specific recognition properties, low cost and short synthesis time. In recent years, novel modified electrodes that are based on molecularly imprinted polymers have had powerful sensor applications in biomolecule/drug detection due to their high selectivity, sensitivity and low toxicity. In this research direction, a highly selective and sensitive molecularly imprinted polymer sensor that is based on hierarchical porous MXene/amino carbon nanotube (MXene/NH_2_-CNT) composites was developed for fisetin detection [86]. As another example, a molecularly imprinted sensor that is based on delaminated titanium carbide MXene and multiwalled carbon nanotubes was also reported for amyloid-β protein recognition and can be used in real samples for clinical applications in Alzheimer’s disease [87].

Immunosensors, which rely on specific antibody–antigen interactions, have also received much attention [88–91]. Salama et al. immobilized bioreceptors (anti-CEA) on ultrathin Ti_3_C_2_T_x_ nanosheets to prepare an immunosensor for label-free, ultrasensitive detection of carcinoembryonic antigen (CEA), which is a cancer biomarker [88]. In this work, Ti_3_C_2_T_x_ was synthesized by minimally intensive layer delamination (MILD) methods and uniformly functionalized with APTES for the covalent immobilization of anti-CEA. The fabricated immunosensor exhibited excellent characteristics with a wide linear detection range of 0.0001–2000 ng·mL^−1^ and a sensitivity of ~ 37.9 µA·ng^−1^·mL·cm^−2^ per decade.

#### Fluorescent/optical biosensors

MXenes possess not only excellent bulk capacitance and metallic conductivity but also superior fluorescence, optical and plasmonic properties, which can be enhanced by surface modification to enhance the properties of MXenes or combining them with other nanomaterials for promising applications in fluorescent biosensors.

MXenes are used as fluorescent quenching agents, which can block the fluorescent signal that is emitted by a fluorescent sensing probe through the interaction of aptamers with MXenes before the detection of target analytes. When the target analytes are added, the aptamer interacts with them and is released, thereby allowing the fluorescence to recover. In 2018, a universal fluorescence resonance energy transfer platform that is based on the Cy3-labeled CD63 aptamer (Cy3-CD63 aptamer)/Ti_3_C_2_T_x_ MXene nanocomplex was developed for the quantitative detection of exosomes [92]. Yang et al. bound Cy3-CD63 aptamers to the surfaces of Ti_3_C_2_T_x_ nanosheets by selective adsorption (through hydrogen bonding and metal chelation) between the aptamer and Ti_3_C_2_T_x_ nanosheets [92]. The fluorescence signal of the Cy3-CD63 aptamer was quenched due to the interaction between Cy3 and the MXenes. Due to the high affinity of the aptamer on the exosome surface with the CD63 protein, the exosome specifically bound to the aptamer with the addition of the exosome, which caused the release of the Cy3-CD63 aptamer from the surfaces of the Ti_3_C_2_T_x_, thereby finally allowing the fluorescence of Cy3 to recover. Meanwhile, the autofluorescence signal of the MXenes showed little change during the whole process and could be used as a standard reference. The self-standard turn-on FRET biosensing system that was developed using this fluorescence-based sensing mechanism is expected to be widely used for the detection of multiple biomarkers.

**Fig. 6:**
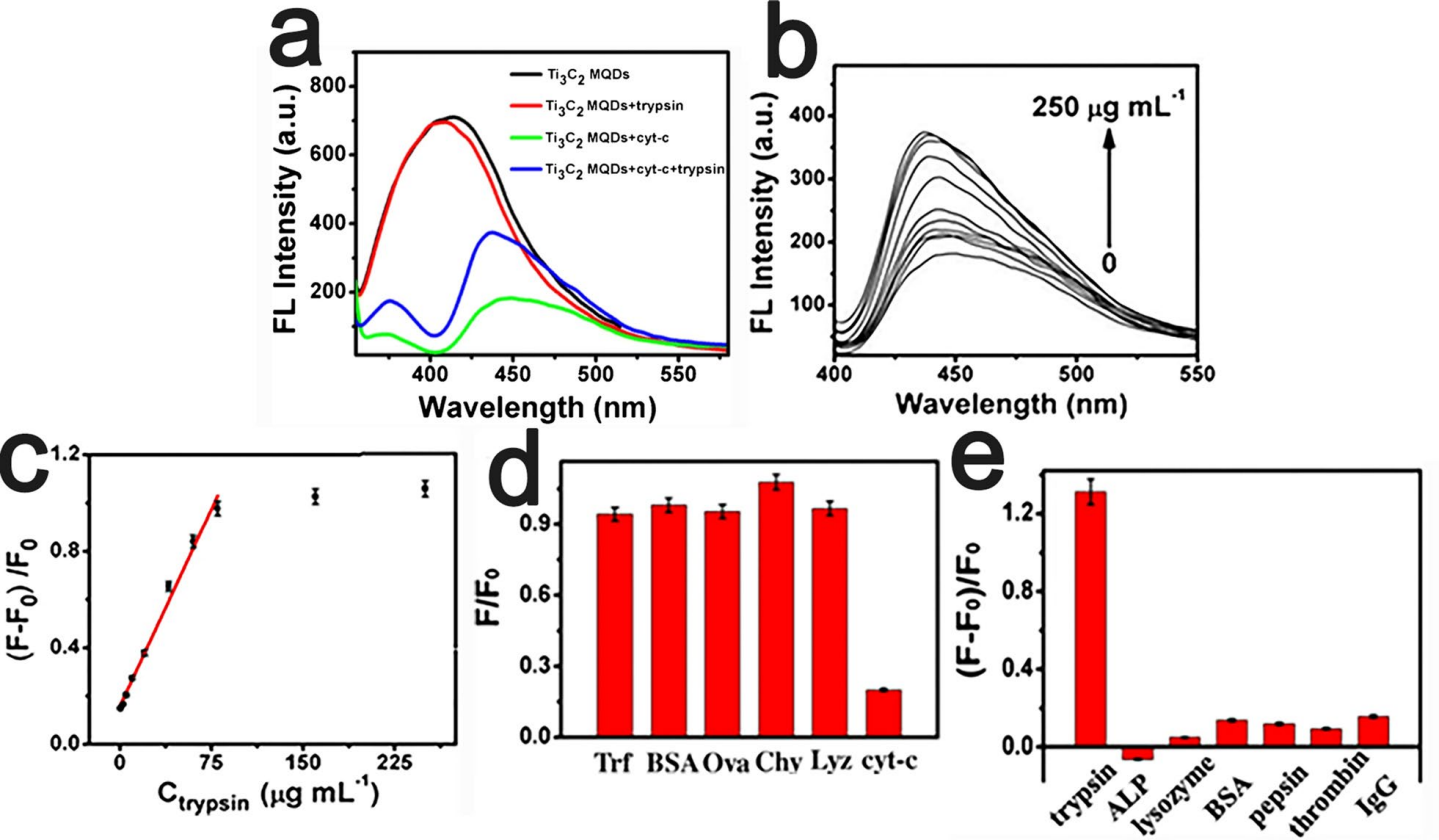
**a** Fluorescence intensity schematic presentation of PLL-protected Ti_3_C_2_ MQDs in 10 mM tris–HCl buffer (*black*); PLL-protected Ti_3_C_2_ MQDs and trypsin (80.0 μg mL^−1^) (*red*); PLL-protected Ti_3_C_2_ MQDs and cyt-c (40.0 μM) (green); PLL-protected Ti_3_C_2_ MQDs, cyt-c (40.0 μM) and trypsin (80.0 μg mL^−1^) (*blue*). **b** Fluorescence intensity schematic presentation of the mixture containing PLL-protected Ti_3_C_2_ MQDs and cyt-c (40.0 μM) in the presence of different concentrations of trypsin (from bottom to top: 0, 0.5, 2.5, 5.0, 10.0, 20.0, 40.0, 60.0, 80.0, 160.0, 250.0 μg mL^−1^. **c** The relationship schematic presentation between the change in fluorescence intensity of the mixture and the trypsin concentration. A linear relationship between changes in fluorescence intensity and trypsin concentration [trypsin] = 0.5, 2.5, 5.0, 10.0, 20.0, 40.0, 60.0, 80.0 μg mL^−1^. *Error bars* represent standard deviations from triplicate measurements. **d** Effect of different proteins (100 μM) on the fluorescence intensity of Ti_3_C_2_ MQDs. e) The selectivity of the PLL-protected Ti_3_C_2_ MQDs toward trypsin using ALP, lysozyme, bovine serum albumin (BSA), pepsin, thrombin and IgG. The concentration of trypsin was 80 μg mL^−1^, and other substances are 200 μg mL^−1^. Reproduced with permission from Ref. [93], © Springer Nature 2019

The excellent photoluminescence (PL) properties of MQDs can also be exploited to develop fluorescent biosensors. For example, an ε-poly-L-lysine (PLL)-decorated Ti_3_C_2_T_x_ MQD-based biosensor was developed for fluorometric determination of cytochrome (cyt-c) and trypsin [93]. The PLL-protected Ti_3_C_2_ MQDs exhibited blue photoluminescence with excitation/emission wavelengths at 330/415 nm and showed a high quantum yield (QY) of the synthesized -PLL-MQDs of approximately 22% due to strong quantum confinement (Fig. 6a). The fluorescence intensity of PL-MQDs decreased with increasing cyt-c content due to the internal filtering effect of cyt-c. Then, with the addition of trypsin, cyt-c was hydrolyzed into small peptides, thereby resulting in the fluorescence intensity of PLL-protected Ti_3_C_2_T_x_ MQDs being restored. The novel and highly sensitive fluorescence turn-off–on nanosensor was successfully applied to the determination of cyt-c and trypsin in spiked serum samples with a low detection limit of 20.5 nm for cyt-c and 0.1 μg/ml for trypsin (Fig. 6).

#### Field-effect transistor

With its hydrophilic surface properties and 2D layered atomic structure, MXene is a promising candidate for the fabrication of biocompatible field-effect transistors (FETs) for fast, direct and label-free detection of biological events[94, 95]. Moreover, MXenes are easily micromachined into various geometries with large contact surfaces, which can greatly simplify the device manufacturing process. A highly sensitive biosensor that is based on ultrathin Ti_3_C_2_T_x_ micrographs was developed by Xu et al. for monitoring dopamine release [94]. The technique that they developed for preparing ultrathin conductive Ti_3_C_2_-MXene transistors is simple and efficient, and the prepared MXene-FET biosensors are compatible with long-term cultured neurons. This study will greatly facilitate the wide application of MXenes in detecting biological events in cellular models. An interdigitated spiral-based MXene-assisted organic electrochemical transistors (isMOECTs) biosensor was developed for the first time for the highly sensitive detection of fPSA / tPSA with an improved detection limit down to 0.01 pg/ml (S/N > 3), demonstrating its potential for clinical diagnosis of human (prostate) cancer, paving a convenient and versatile platform for detect other biomarkers in various types of cancer or for liquid biopsy [96].

Due to their unique laminar morphology, excellent biocompatibility and superior electrical properties, Mxenes have been used in biosensors such as electrochemical biosensors, fluorescent biosensors and immunosensors, which provides a large-area immobilization for the inclusion of biological recognition elements such as enzymes, molecularly imprinted polymers and nucleic acids. These MXene-based biosensors have proven to have excellent performance parameters such as low lower detection limits, high sensitivity, short response times and a wide linear range. As high-performance receptors, they have high selectivity, a low limit of detection (LOD), high sensitivity, a short response time, and a wide linear range, which are the main performance parameters.

### Diagnosis

In addition to their promising applications in biosensors, MXene-based materials also play a significant diagnostic role. Imaging technology is indispensable for the early diagnosis of cancer and is very important for the precise localization and staging of tumors and for guiding cancer treatment and detecting cancer recurrence after treatment. The excellent physicochemical properties of MXene nanosheets give them great potential for diagnostic imaging, and they can be applied with various imaging techniques, such as X-ray computed tomography (CT), magnetic resonance imaging (MRI), photoacoustic imaging and fluorescent imaging. Imaging techniques that are based on novel MXene-based reagents are beneficial for overcoming some of the common problems and drawbacks of current reagents. For example, compared to conventional imaging agents, 2D MXene-based reagents have quantum size effects for photoluminescence (PL) cell imaging, which can enhance the intrinsic photothermal properties for PA imaging and elemental contrast for X-ray CT imaging.

#### Luminescent imaging

**Fig. 7:**
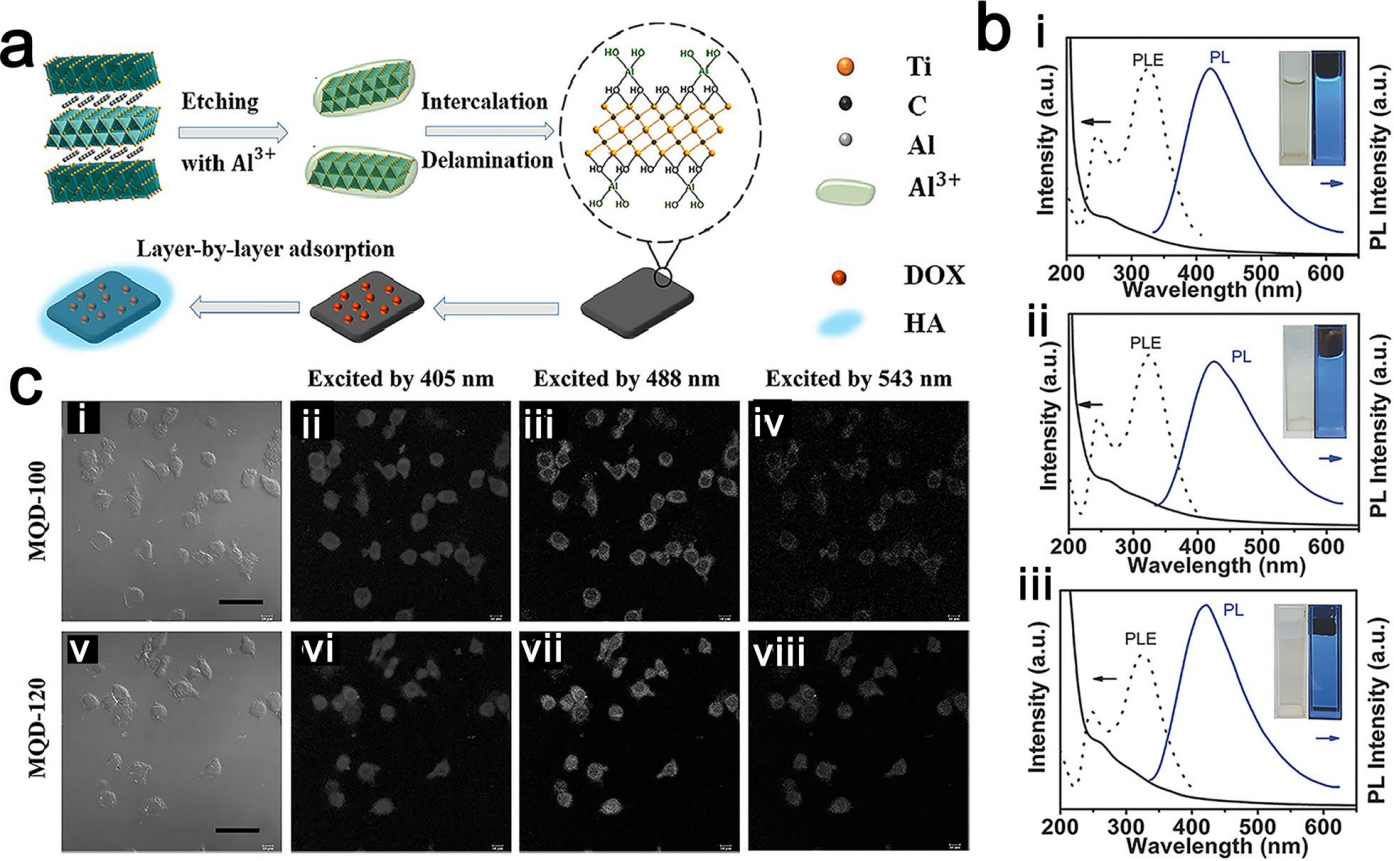
**a** Schematic diagram of the preparation of multifunctional nanoplatform (Ti_3_C_2_-DOX). Reproduced with permission from Ref. [98], © American Chemical Society 2017. **b** UV–Vis spectra (solid line), PLE (dashed line) and PL spectra (solid line, Ex = 320 nm) of MQD-100 (i), MQD-120 (ii) and MQD-150 (iii) solutions under visible light and 365 nm UV lamp. Reproduced with permission from Ref. [58], © John Wiley and Sons 2017. **c** (i)(v) Bright-field imaging of RAW264.7 cells. Confocal imaging (405, 488, and 543 nm) of RAW264.7 cells incubated with (ii–iv) MQD-100 and (vi–viii) MQD-120. Reproduced with permission from Ref. [58], © John Wiley and Sons 2017

Conventional MXene-based materials exhibit extremely low luminescence properties in aqueous solutions, in which no photoluminescence response can be detected. To further broaden their biomedical applications, researchers have used various strategies to enhance the fluorescence properties of MXenes. There are currently two main ways in which the fluorescence properties of MXene materials can be enhanced. One of the strategies is attaching a fluorescent species to the surfaces of MXenes to equip them with fluorescent properties. Liu et al. loaded the cationic fluorescent drug DOX onto an MXene with the p-terminus aluminum oxide anion by electrostatic adsorption to obtain coupling (Fig. 7a) [98]. Due to the autofluorescence effect of the anticancer drug DOX, this system can be used for biological imaging as well as anticancer therapy.

Another strategy is to prepare MXene quantum dots (MQDs) with luminescence properties. Similar to graphene quantum dots (QDs) [99], molybdenum disulfide QDs [100] and boron nitride QDs [101], MQDs [54, 102] exhibit excitation-dependent luminescence properties and, thus, have potential applications in efficient fluorescence imaging [103]. MXene flakes can be broken into quantum dots, which have extremely small sizes and excellent photoluminescence (PL) properties, by a variety of methods, including hydrothermal methods [55, 60, 104]. Compared with conventional organic fluorescein, quantum dots of inorganic two-dimensional nanomaterials, including MQDs, have the advantages of tunable wavelength, high chemical stability and photostability, high photoluminescence quantum yield, low cytotoxicity, and high dispersibility for bioimaging, which can be tuned by changing the size, shape, or functionality of the prepared quantum dots. Although strong photoluminescence effects can be observed in 2D materials such as graphene and MXene quantum dots, there is still controversy about the mechanism of their luminescence. There are two main views on the mechanism of luminescence in 2D materials: size effect and surface defects [105, 106]. In 2016, Xue et al. prepared monolayered Ti_3_C_2_T_x_ QDs at temperatures of 100 °C (MQD-100), 120 °C (MQD-120) and 150 °C (MQD-150) by a facile hydrothermal method and demonstrated that these MQDs had excitation-dependent luminescence properties [58]. From the UV–vis spectra and the PL excitation (PLE) spectrum that was recorded with the strongest luminescence, they concluded that the MQDs exhibited excitation-related PL behavior (Fig. 7b). By studying the variation of PL intensities of MQDs at various pH values, it was found that MQDs are stable enough to be used in situations with various pH values. Preliminary studies on MQD-100 and MQD-120 cell imaging were performed by labeling RAW264.7 cells, which demonstrated the great potential of MQDs as biocompatible multicolor cell imaging reagents [58] (Fig. 7c).

**Fig. 8:**
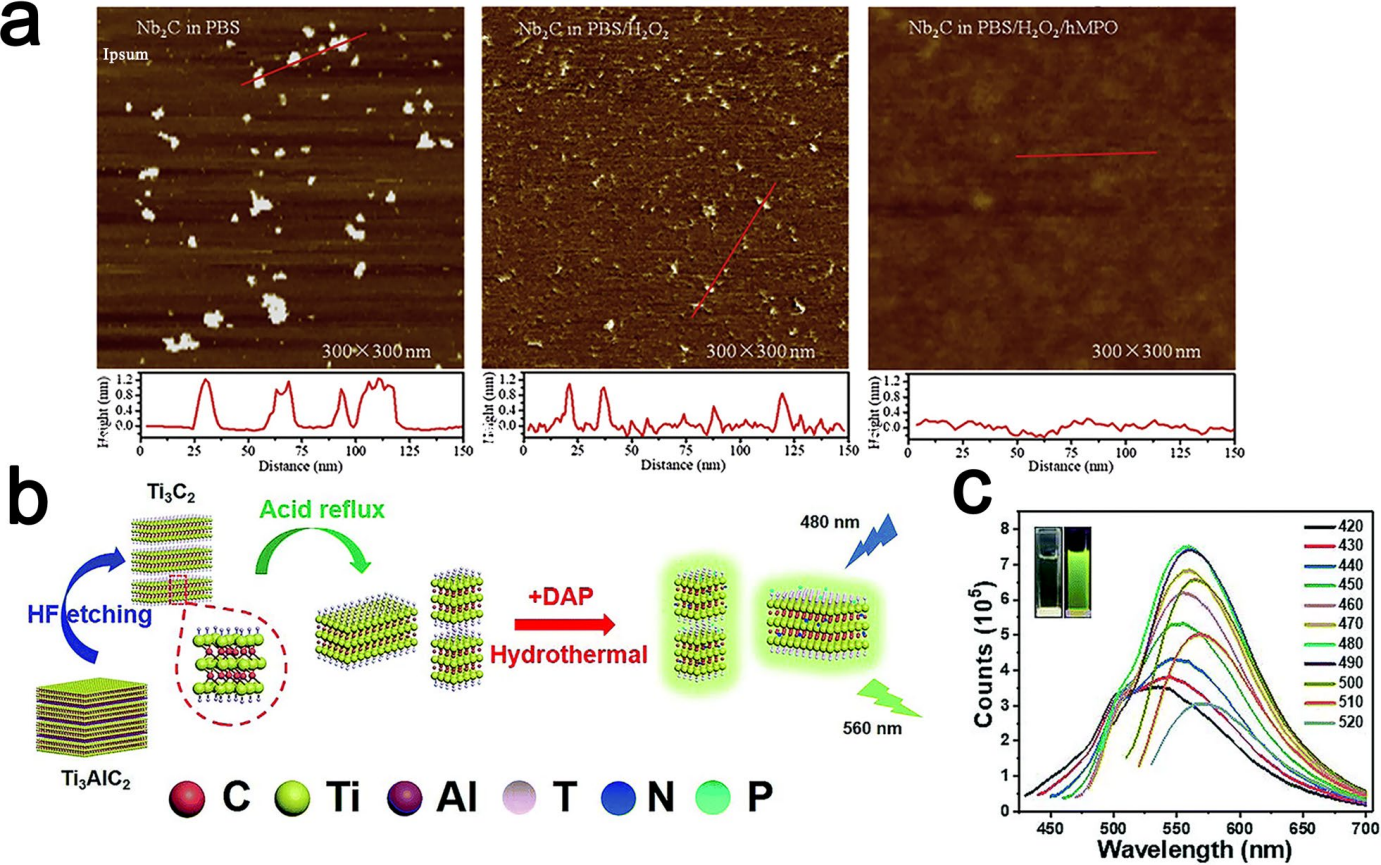
**a** The AFM images of Nb_2_CT_x_ quantum dots after 24 h of different biodegradation treatments and the corresponding height distributions. Reproduced with permission from Ref. [107], © Elsevier 2020. **b** Schematic diagram of the preparation of N,P-MQD [22]. **c** Fluorescence emission spectra of the N, P-MQDs prepared at 120 ℃ at different excitation wavelengths. Inset: photo under UV light (365 nm). Reproduced with permission from Ref. [22], © Royal Society of Chemistry 2019

Although inorganic nanofluorophore fluorescence has good potential for imaging applications due to its satisfactory biological properties, a nonnegligible disadvantage of these inorganic nanofluorophores is that they are usually nonbiodegradable [18]. Yang et al. synthesized Nb_2_CT_x_ QDs in tetrapropylammonium hydroxide (TPAOH) solution using ultrasound-assisted physicochemical exfoliation [107]. Compared with conventional nanofluorescence, the prepared Nb_2_CT_x_ QDs exhibited excellent chemical stability and biocompatibility, especially surprising enzyme-responsive biodegradability and excellent antiphotobleaching ability (Fig. 8a).

As the utilization of MQDs becomes increasingly widespread, the modification strategies of MQDs are increasingly being investigated. The properties of MQDs can be further improved by suitable modification to improve their performance for cell imaging. The electronic properties and structure of quantum dots can be significantly changed by doping with N, Cu, and P, among other elements, to realize higher quantum yields (QY), better stability and more surface active centers [108–110]. Guan et al. prepared nitrogen-phosphorus functionalized Ti_3_C_2_T_x_ MXene-based quantum dots (N,P-MQDs) by a top-down hydrothermal method, which greatly increased the photoluminescence quantum yield (PLQY) to 20.1% (Fig. 8b) [22]. Moreover, the prepared photoluminescent quantum dots exhibited strong green fluorescence near 560 nm under 480 nm excitation for the first time (Fig. 8c). As another example, Lu et al. synthesized a class of N-Ti_3_C_2_T_x_ quantum dots using two-dimensional Ti_3_C_2_T_x_ as the raw material, along with DMF as the solvent medium and nitrogen-doping agent simultaneously [111]. The obtained N-Ti_3_C_2_T_x_ quantum dots showed good dispersion stability and were further compounded with DAP to form a composite nanoprobe (N-Ti_3_C_2_T_x_ quantum dots@DAP nanoprobe).

In addition to these two strategies, researchers have developed other methods for applying MXenes to bioimaging. Wang et al. synthesized ultrasmall MXenes with monolayer thickness, lateral dimensions of 2–8 nm, and bright and tunable fluorescence by simultaneous layer cutting and stacking cleavage in aqueous TMAOH solution using a solvothermal approach [112]. Moreover, Zhou et al. innovatively developed a method for the synthesis of graphene quantum dots (GQDs) from layered Ti_3_C_2_Tx by solvent heat treatment of Ti_3_C_2_T_x_ in dimethylformamide (DMF) [113].

Researchers improve the fluorescent properties of MXenes through the strategy of loading fluorescent species on the MXenes surface and preparing MQDs with luminescent properties, confirming the potential of MXenes for fluorescent imaging. With the development of MQDs modification strategies, the performance of MQDs for cellular imaging continues to be improved. Meanwhile, more methods for applying MXenes to bioimaging are being developed.

#### Photoacoustic imaging (PAI)

PAI is a noninvasive and nonionizing biomedical imaging technique that has emerged in recent years. A nonionizing pulsed laser is irradiated onto biological tissues and converted into ultrasound waves (also called photoacoustic signals) by the light absorbing domains on the tissues. The photoacoustic signal, which carries information about the light absorption characteristics of the tissue, is accepted by the ultrasonic transducer and transformed into an image of the light absorption distribution of the tissue. Compared to pure optical tissue imaging, PAI principally avoids the effects of light scattering and provides a higher spatial distribution rate for living objects, thereby enabling deeper tissue imaging [114, 115]. Therefore, an effective PAI contrast agent should have excellent photothermal conversion ability to produce a signal that is in significant contrast to the PA signal that is formed by the surrounding tissue. MXene nanosheets with the LSPR effect are considered to be very attractive PAI contrast agents. A variety of MXenes, including Ti_3_C_2_T_x_ [116, 117], Nb_2_CT_x_ [18], and Ta_4_C_3_T_x_ [71]_,_ have been reported to have excellent photothermal conversion properties.

Ta_4_C_3_T_x_-SP demonstrates good potential for use in PA contrast agents due to its satisfactory photothermal conversion efficiency and biocompatibility. The extinction coefficient (ε) and photothermal conversion efficiency (η) are the two main parameters that determine the photothermal performance of a photothermal converter. The extinction coefficient reflects the absorption capacity of light while the photothermal conversion efficiency reflects the performance of the photothermal converter. Two-dimensional ultrathin Ta_4_C_3_T_x_ nanosheets that were prepared by the liquid-phase exfoliation method, which combines HF etching and probe ultrasonication, possessed excellent near-infrared photothermal properties, with an extinction coefficient of 4.06 lg^−1^·cm^−1^ at 808 nm, a photothermal conversion efficiency of 44.7%, and good photothermal stability [71]. Moreover, the surface modification of Ta_4_C_3_T_x_ nanoflakes with biocompatible soybean phospholipids greatly improved the biocompatibility and physiological stability of Ta_4_C_3_ nanoflakes, and in vitro and in vivo tests did not show any noticeable toxicity [71]. Lin et al. also experimentally demonstrated the use of Nb_2_CT_x_-PVP (polyvinylpyrrolidone) for PAI, with an extraordinarily high photothermal conversion efficiency (36.4% at NIR-I and 45.65% at NIR-II) and high photothermal stability [18].

Of course, in addition to applications in fluorescence imaging, MQDs, which are characterized by a strong and broad near-infrared absorption band, are also ideal imaging agents for tumor PAI. In 2019, an MQD was prepared by a fluorine-free method. Due to the modification of a large amount of aluminum oxygen anions on its surface, the quantum dots exhibited stronger and wider absorption capabilities in the near-infrared region with an extinction coefficient of as high as 52.8 lg^−1^·cm^−1^ at 808 nm and a photothermal conversion efficiency of as high as 52.2%. The prepared MXene quantum dots achieved simultaneous photoacoustic (PA) imaging and PTT effects on tumors [118]. Overall, due to its low tissue attenuation coefficient, MXene-based PAI can hopefully overcome the penetration limitations of traditional optical imaging techniques to achieve deeper tissue imaging as a promising imaging tool.

#### Computed tomography (CT) imaging

CT imaging is one of the most widely used and effective diagnostic imaging tools due to its high spatial resolution, noninvasiveness and deep tissue penetration. CT imaging is based mainly on the variability of tissue absorption of rays, and one section after another of a body part is scanned to form a 3D image [119, 120]. Nanomaterials that contain high atomic number elements such as bismuth, cesium, tantalum and tungsten are often considered potential CT imaging materials due to their ability to attenuate X-rays [120]. The most common clinically approved CT contrast agents, namely, iodine-containing compounds, have been shown to be inappropriate for patients who require repeat CT scans or are at high risk due to their short circulation time in the bloodstream and high toxicity [121, 122]. Therefore, the search for CT imaging agents with high atomic number elements and better biocompatibility is a popular direction in the development of CT. Two-dimensional materials such as MXenes have attracted much attention from researchers in the biomedical field due to their unique physicochemical properties and structural characteristics.

**Fig. 9:**
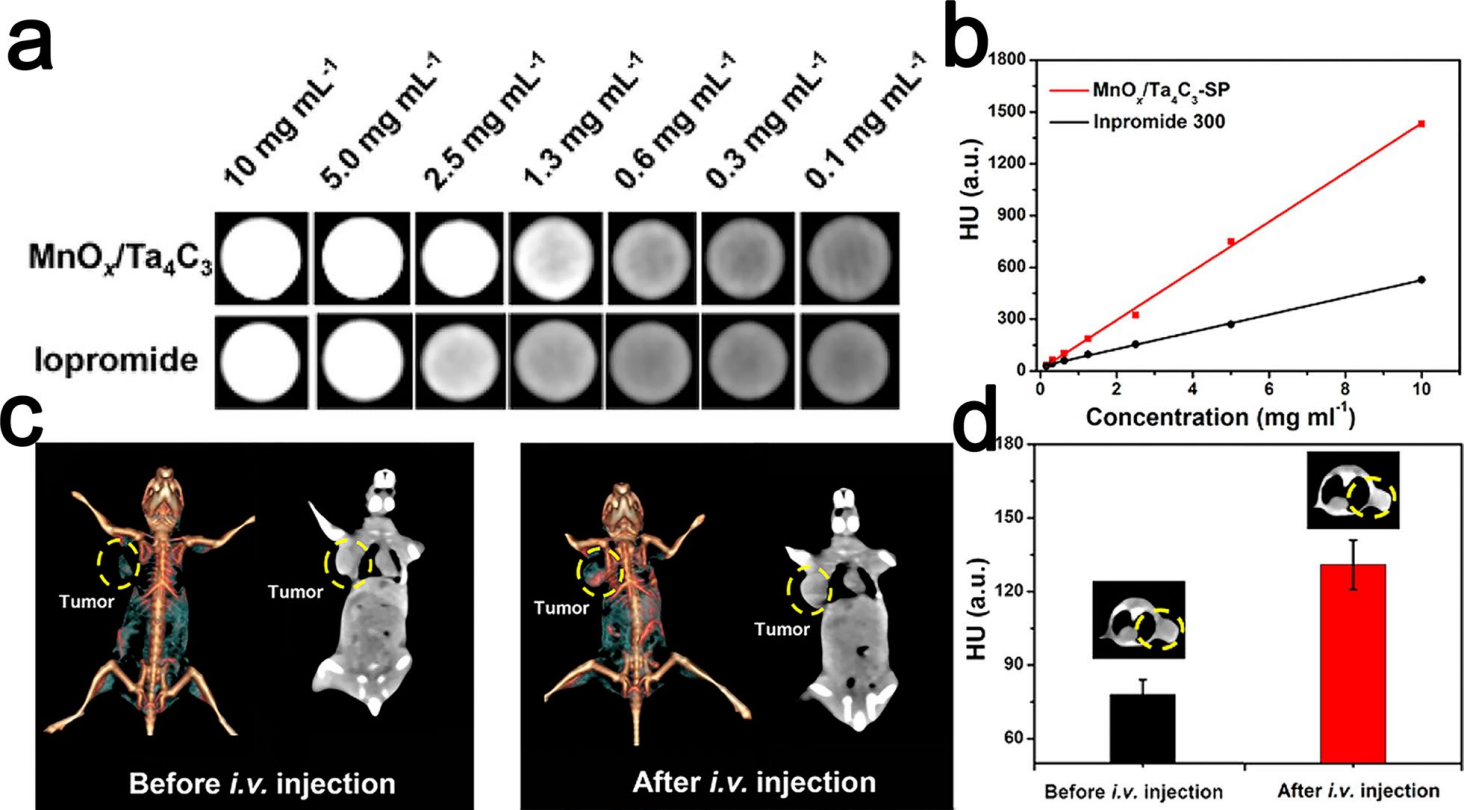
In vitro CT images (**a**) and HU values (**b**) of MnO_x_/Ta_4_C_3_T_x_-SP composite nanosheet solution and iopromide solution with varied concentrations (concentration of Ta, I). **c** In vivo 3D reconstructed CT (left) and contrast (right) images of mice before and after intravenous injection of MnO_x_/Ta_4_C_3_T_x_-SP composite nanosheets (20 mg·kg.^−1^, 100 μL) for 2 h. **d** CT comparison of tumor tissues in vivo before and after intravenous administration of MnO_x_/Ta_4_C_3_T_x_-SP composite nanosheets. Reproduced with permission from Ref. [117], © American Chemical Society 2017

Tantalum (Ta) is an element with a high atomic number (Z = 73) and a high X-ray attenuation coefficient. At 100 eV, the X-ray attenuation coefficient of tantalum is 4.3 cm^2^/kg, compared to 5.16 cm^2^/kg for gold [123]. Ta-based MXenes Ta_4_C_3_T_x_ are considered to be ideal agents for CT imaging [71, 117, 124]. The brightness and corresponding enhanced Hounsfield unit (HU) values of CT images of MnO_x_/Ta_4_C_3_T_x_-SP composite nanosheets showed a good linear positive correlation with the concentration of Ta, which was enhanced with increasing Ta concentration (Fig. 9a, b). Compared with the CT imaging effect of iodine-based iopromide, which is currently used in clinical practice, in vitro CT images of MnOx/Ta_4_C_3_T_x_-SP composite nanosheets showed a stronger signal and higher contrast at the same elemental concentration (Fig. 9c, d) [117].

#### Magnetic resonance imaging (MRI)

MRI, which is another noninvasive clinical imaging modality, has similar imaging capabilities to CT, which requires the use of harmful rays [125]. However, MRI technology shows the structure of human soft tissues more clearly, can directly obtain native 3D cross-sectional images without reconstruction, and causes no damage to the body with ionizing radiation [126]. Gadolinium(III) complexes are now widely used and typical MRI contrast agents. However, their toxicity to the kidney has been of increasing concern in recent years. The use of gadolinium(III)-based MRI contrast agents in patients with renal failure is likely to result in fatal nephrogenic systemic fibrosis (Nsf), and metallic gadolinium deposits have recently been observed in the brains of patients with normal renal function [127, 128]. Therefore, finding an MRI contrast agent with high biosafety and low toxicity to improve the quality and specificity of MRI has attracted much attention from the biomedical community.

**Fig. 10:**
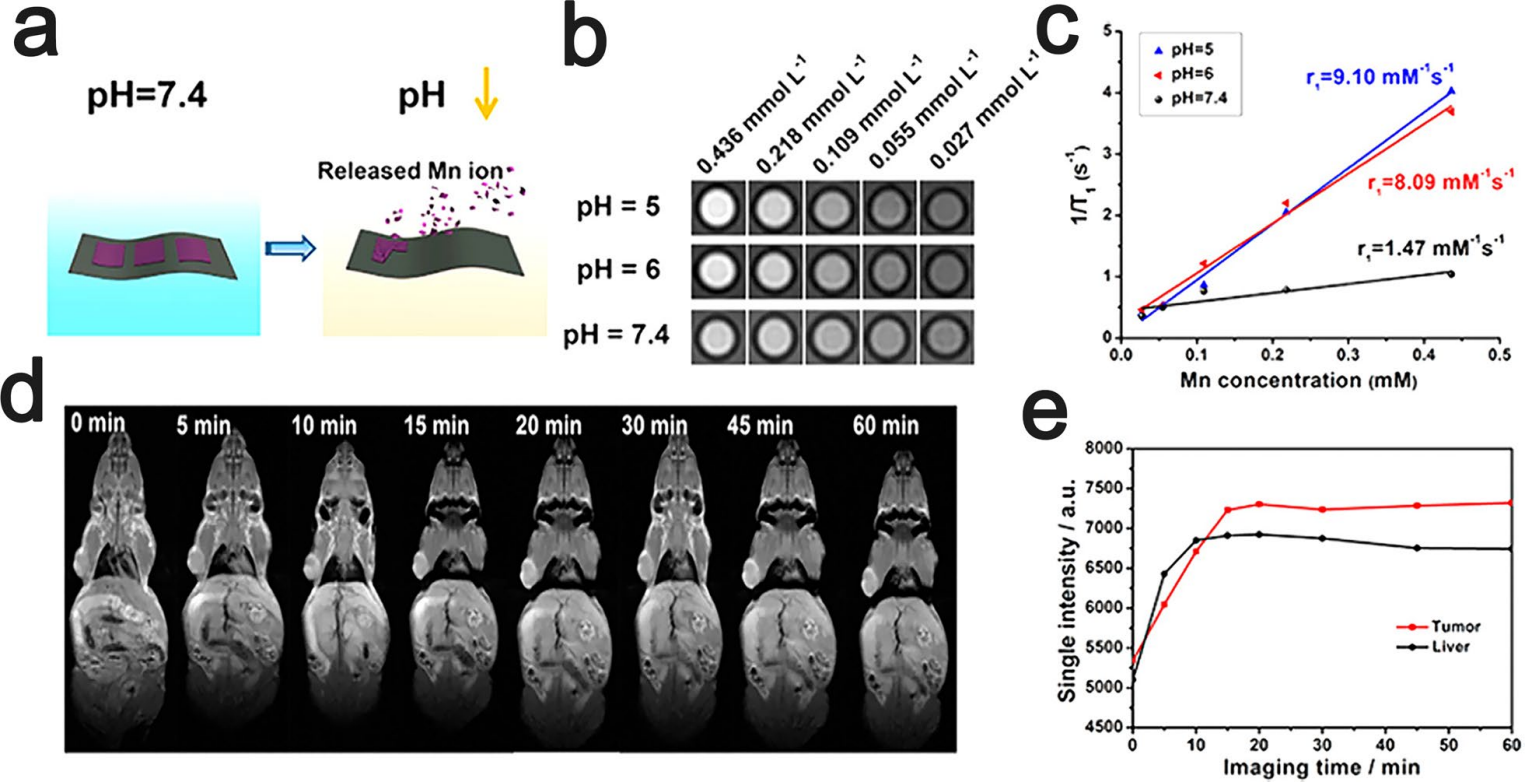
Contrast-enhanced pH-responsive MRI of MnO_x_/Ti_3_C_2_T_x_-SP nanosheets in vitro and in vivo. **a** Schematic diagram of the disintegration of the MnO_x_ fraction under weakly acidic conditions. **b** In vitro T1-weighted magnetic resonance imaging of MnO_x_/Ti_3_C_2_T_x_-SP nanosheets after 3 h immersion in buffered solutions with different pH values. **c** MnO_x_/Ti_3_C_2_T_x_-SP nanosheets soaked in buffer solutions of different pH values for 3 h, 1/T1 vs. Mn concentration. T1-weighted imaging **d** and detection of the corresponding MRI signal intensity **e** after intravenous injection of MnO_x_/Ti_3_C_2_T_x_-SP composite nanosheets to mice at different time points were performed. Reproduced with permission from Ref. [116], © American Chemical Society 2017

As a novel biocompatible material, manganese (Mn)-based paramagnetic agents have great potential for clinical applications in magnetic resonance imaging [116, 129]. For instance, paramagnetic MnO_x_ was firmly immobilized on the surfaces of Ti_3_C_2_T_x_ nanosheets by the "redox reaction-induced growth" (RR-IG) method, and the stability of the MnO_x_/Ti_3_C_2_T_x_ composite nanosheets (MnO_x_/Ti_3_C_2_T_x_-SP) was greatly improved by further modification of the surface with soy phospholipids (SP) [116]. Since the surface-anchored paramagnetic MnO_x_ component shows unique pH-responsive T1-weighted MRI capability, the prepared MnO_x_/Ti_3_C_2_T_x_-SP composite nanosheets can be used for MRI of tumors. The Mn–O bond is easily broken under the mildly acidic microenvironment of the tumor to release Mn^2+^ ions (Fig. 10a), which maximizes the opportunity for interaction between paramagnetic Mn centers and water molecules, thereby further improving the T1-weighted MRI performance [130]. This conclusion was supported by in vitro experiments, in which the enhancement of MRI signal (Fig. 10b, c) and the dissociative release of Mn^2+^ under acidic conditions (Fig. 10a) were observed, and in vivo experiments in mice, in which the MRI signal was significantly enhanced in tumors (Fig. 10d, e) [116]. To further evaluate the capability of MnO_x_/Ti_3_C_2_T_x_-SP composite nanosheets, T1-weighted imaging was performed at various times after intravenous administration of a suitable dose of MnO_x_/Ti_3_C_2_T_x_-SP composite nanosheets (dose: 2 mg·mL^−1^, 100 μL) to mice. In the results of T1-weighted imaging, a significant brightening effect of MRI signals in tumors was observed, and the signals were gradually enhanced with prolonged imaging duration (Fig. 10d, e) [116].

**Fig. 11:**
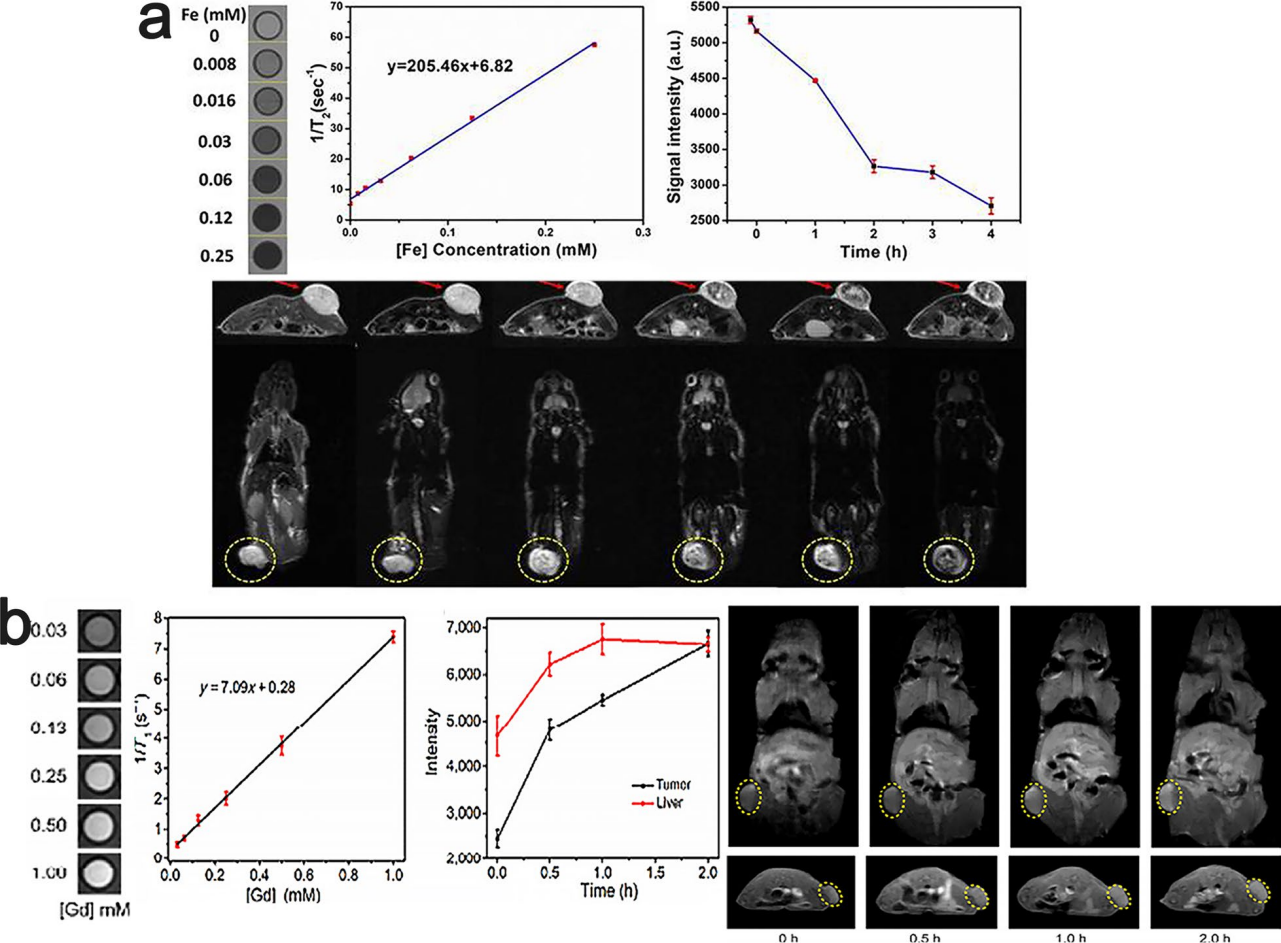
**a** Transverse and coronal section of T_2_-weighted MRI of 4T1 tumor-bearing mouse before and after intravenous injection of Ta_4_C_3_T_x_-IONP-SPS at different time points. Regions of hypointense signal T_2_ images found at the tumor site became more obvious as the observation time increased. Reproduced with permission from Ref. [124], © Ivyspring International Publisher 2021. **b** In vivo MRI signal intensity of a tumor and liver of 4T1 tumor-bearing mice after i.v. administration. Reproduced with permission from Ref. [131], © Springer Nature 2018

On the other hand, IONPs have been widely investigated as an effective contrast agent for MR imaging. Attempts to prepare agents for multimodal imaging were also performed by Liu and his coworkers, who successfully immobilized superparamagnetic iron oxide nanoparticles (IONPs) on the surface of a 2D MXene, namely, Ta_4_C_3_T_x_, by in situ growth to produce Ta_4_C_3_T_x_-IONP, thereby endowing the Ta_4_C_3_T_x_/superparamagnetic iron oxide (IONP) nanocomposite with contrast-enhanced T_2_-weighted MR imaging capability [124] (Fig. 11a). A different strategy was reported by Zong and colleagues, who prepared GdW_10_@Ti_3_C_2_ composites by depositing GdW_10_ onto the surfaces of Ti_3_C_2_ nanoflakes, which can be used as contrast agents for enhanced CT and MR imaging [131] (Fig. 11b).

Due to its high biocompatibility and unique physicochemical properties, MXene has been shown to have potential to be used as an imaging agent of CT, PAI and MRI imaging for diagnostic imaging and additional studies have further advanced the clinical application of MXenes.

### Therapy

Due to their excellent physicochemical properties and unique structural characteristics, MXenes have been applied in various fields of biomedicine. To date, in addition to biosensor and diagnostic applications, various types of MXenes and their composites have been developed for therapeutic applications, including drug delivery systems, typical photothermal therapy (PTT), photodynamic therapy (PDT), immunotherapy and synergistic combinations of multiple technologies for treatment.

#### Drug delivery systems

Due to the distinctive structure of MXenes, MXenes can be applied to construct gene/drug delivery systems to further achieve targeted drug delivery, reduce drug toxicities and improve the pharmacokinetics of drug molecules. The nanoscale size of MXene materials facilitates intravenous delivery to and efficient accumulation at the diseased site during the treatment process. Moreover, the two-dimensional planar topology endows MXenes with their characteristic large specific surface area, thereby providing abundant sites for therapeutic molecules to anchor on the surface of the laminar structure. Currently, cancer is a major disease that threatens human health and causes many deaths worldwide every year. MXene-based materials can effectively attack cancer cells through controlled drug release and enhancement of the cellular uptake of the payload [132–135]. MXenes are considered effective anticancer tools based on preliminary studies.

**Fig. 12:**
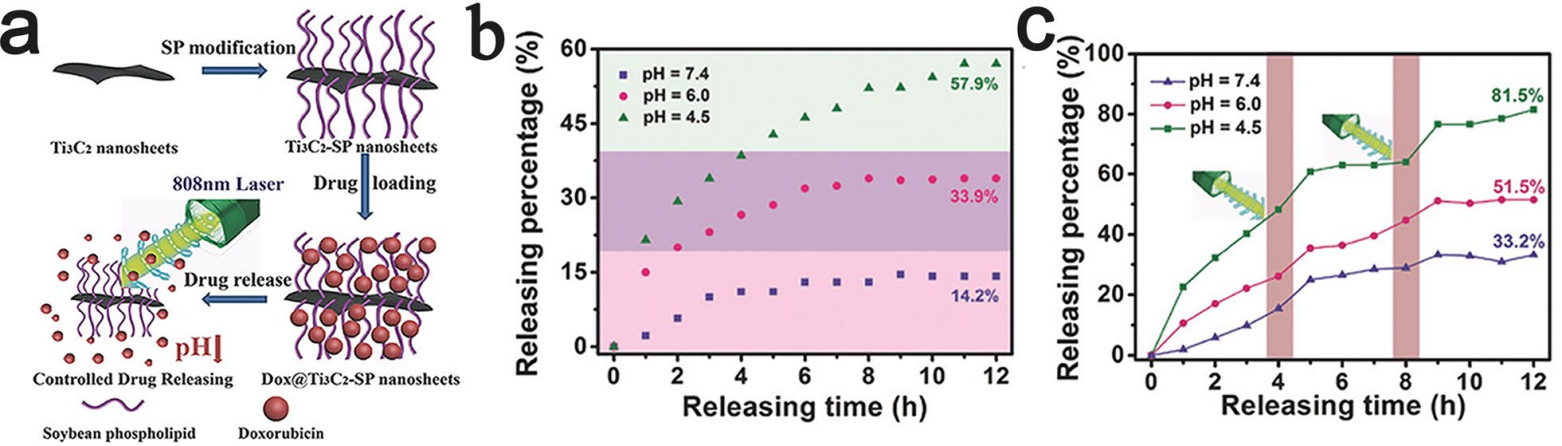
**a** Schematic diagram of the pH-responsive and laser-triggered drug release of DOX-loaded Ti_3_C_2_T_x_-SP nanosheets [137]. **b** The Dox release profiles of Dox@Ti_3_C_2_T_x_-SP nanosheets in buffer solutions at different pH values [137]. **c** The Dox release profiles triggered by 808 nm laser irradiation at different pH values. Reproduced with permission from Ref. [137], © John Wiley and Sons 2018

Chen et al. established a multifunctional Ti_3_C_2_-based nanoplatform (Ti_3_C_2_T_x_-DOX) via layer-by-layer surface modification of doxorubicin (DOX) and hyaluronic acid (HA), which was achievable due to the negative charge of tumor-targeted hyaluronic acid (HA) and the surface of Ti_3_C_2_T_x_ and the positive charge of DOX [136]. Ti_3_C_2_T_x_ that were synthesized by tetrapropylammonium hydroxide (TPAOH) intercalation were functionalized with hydroxyl groups, which further enhanced the photothermal performance and light harvesting capability in the NIR region. HA coating of the outer layer of the nanosheets improved the biocompatibility of the system and enabled active targeting of tumor cells via CD44^+^ overexpression on the cell membranes of cancer cells. This Ti_3_C_2_T_x_-DOX showed a drug loading capacity of as high as 84.2%. Moreover, in vitro and in vivo experiments showed that the system could exhibit excellent biocompatibility and efficient pH-responsive and NIR laser-induced drug-releasing behavior [136]. Similar to the abovementioned study, anticancer drugs (doxorubicin, DOX) can also be loaded onto the surfaces of SP-modified Ti_3_C_2_ nanosheets with a large specific surface area (Ti_3_C_2_T_x_-SP) for highly efficient tumor eradication (Fig. 12a). Ti_3_C_2_T_x_-SP, which is a novel drug-delivery nanosystem, also features high drug-loading capacity (up to 211.8%), pH responsiveness (Fig. 12b and NIR laser-triggered drug release (Fig. 12c) [137].

However, MXenes lack a confined space for high loading of drugs, which is a possible challenge for the use of MXenes as drug-delivery carriers. To enhance drug loading/release capabilities and extend the biomedical applications of MXenes, surface nanopore engineering of Ti_3_C_2_T_x_ was performed based on facile sol–gel chemistry in a recent study [138]. The surface of Ti_3_C_2_T_x_ was successfully coated with a thin mesoporous silica shell layer under alkaline synthesis conditions using cetyltrimethylammonium chloride (CTAC) as a mesoporous guide and tetraethylorthosilicate (TEOS) as a precursor (Ti_3_C_2_T_x_@mMSNs), which improved the interfacial properties of Ti_3_C_2_T_x_ and combined the advantages of both materials as drug carriers, including a space-confined mesoporous structure, enhanced hydrophilicity, suitable surface chemistry and dispersibility. To achieve an active targeting response to the tumor region, arginine-glycine-aspartic acid (RGD) was bound to polyethylene glycol (PEG)-modified Ti_3_C_2_T_x_@mMSNs by covalent interaction. The prepared Ti_3_C_2_T_x_@mMSNs have a good mesoporous structure with a uniform pore size (3.1 nm), high pore volume (0.96 cm^3^/g) and large specific surface area (772 m^2^/g). In vitro and in vivo evaluations have shown that the novel MXene-based composite nanosystems that are synthesized by this method have high active-targeting capability and biocompatibility and can completely eradicate tumors without significant recurrence by synergizing with conventional chemotherapy and photothermal hyperthermia [138].

In addition, a promising post-MXene materials, MBene, has recently been developed as a multifunctional nano-delivery platform. He et al. successfully synthesized the 2D zirconium boride nanosheet (ZBN) by a microwave-associated chemical etching method, which has excellent NIR-photothermal property with a high photothermal conversion efficiency of 76.8% in the NIR-II window (1060 nm) and obtain good dispersion through surface modification of hyaluronic acid (HA) by borate esterfication. High drug loading (ZBN-HA/DOX and ZBN-HA/NO) was achieved by loading doxorubicin (DOX) and NO prodrug (Gal-NO) on the surface of ZBN-HA via borate esterification. the photopyrolysis of ZBN-HA/DOX and ZBN-HA/NO allowed HA deconjugation and ZBN aggregation, realizing photocontrolled intratumoral retention and drug release [139].

In conclusion, MXenes are already an ideal drug carrier due to their nano-size and two-dimensional planar topology.

#### Photothermal therapy

For the treatment of cancer, which is one of the most dangerous diseases to human health, traditional therapeutic strategies mainly include surgery, chemotherapy and radiation therapy. However, surgery alone usually does not completely remove all cancerous tissues, and radiotherapy kills cancer cells while having a greater toxic effect on normal tissues and cells. In recent years, emerging photothermal therapy (PTT) has attracted much attention for its excellent performance in cancer treatment. A nanomaterial with photothermal activity, namely, a photothermal agent (PAT), is delivered to the cancer site without damaging the surrounding healthy tissue. Due to the poor heat resistance of tumor cells, the photothermal agent can convert near-infrared light into heat energy at the tumor site to generate superheat, which leads to a series of hazards, such as protein denaturation, cell lysis, and organelle damage, thereby killing cancer cells [140, 141]. The ideal photothermal agent has high selectivity for the target tissue, a large absorption cross section for optical wavelengths, low toxicity, and easy functionalization [142]. Various nanomaterials have been reported for PTT, such as gold nanorods [143, 144], copper sulfide nanoparticles [145], and black phosphorus [146]. MXenes, including Ti_3_C_2_T_x_ [147, 148], Nb_2_CT_x_ [18, 149] and Ta_4_C_3_T_x_ [141], have also become new PTT reagents for deep tissues due to their remarkable photothermal conversion efficiency and strong absorption in the near-infrared wavelength range, and the use of MXenes has been successfully demonstrated for in vivo PTT.

In recent years, researchers have made a series of breakthroughs in the development of MXene materials for PTT, including Nb_2_C with extremely high photothermal conversion efficiency in both the NIR-I and NIR-II regions [18, 149], Ta_4_C_3_T_x_-SP nanosheets that enable dual-mode CT and PA imaging of living tumors [117], and MQDs with extremely high photothermal conversion efficiency [118, 150]. Ti_3_C_2_ material was synthesized through rational design with the (MnO)x composition anchored on the surface of Ti_3_C_2_ by redox reaction by Dai et al. [116]. To further improve the stability of (MnO)_x_/Ti_3_C_2_T_x_, the surface was also modified with soy phospholipids (SP). After experimental validation, the synthesized MnO_x_/Ti_3_C_2_T_x_-SP composites showed much higher photothermal stability and achieved a photothermal conversion efficiency of 22.9%, which is comparable to those of conventional Au nanorods (21%) and Cu_2-x_Se carbon nanotubes (22%) [116].

**Fig. 13:**
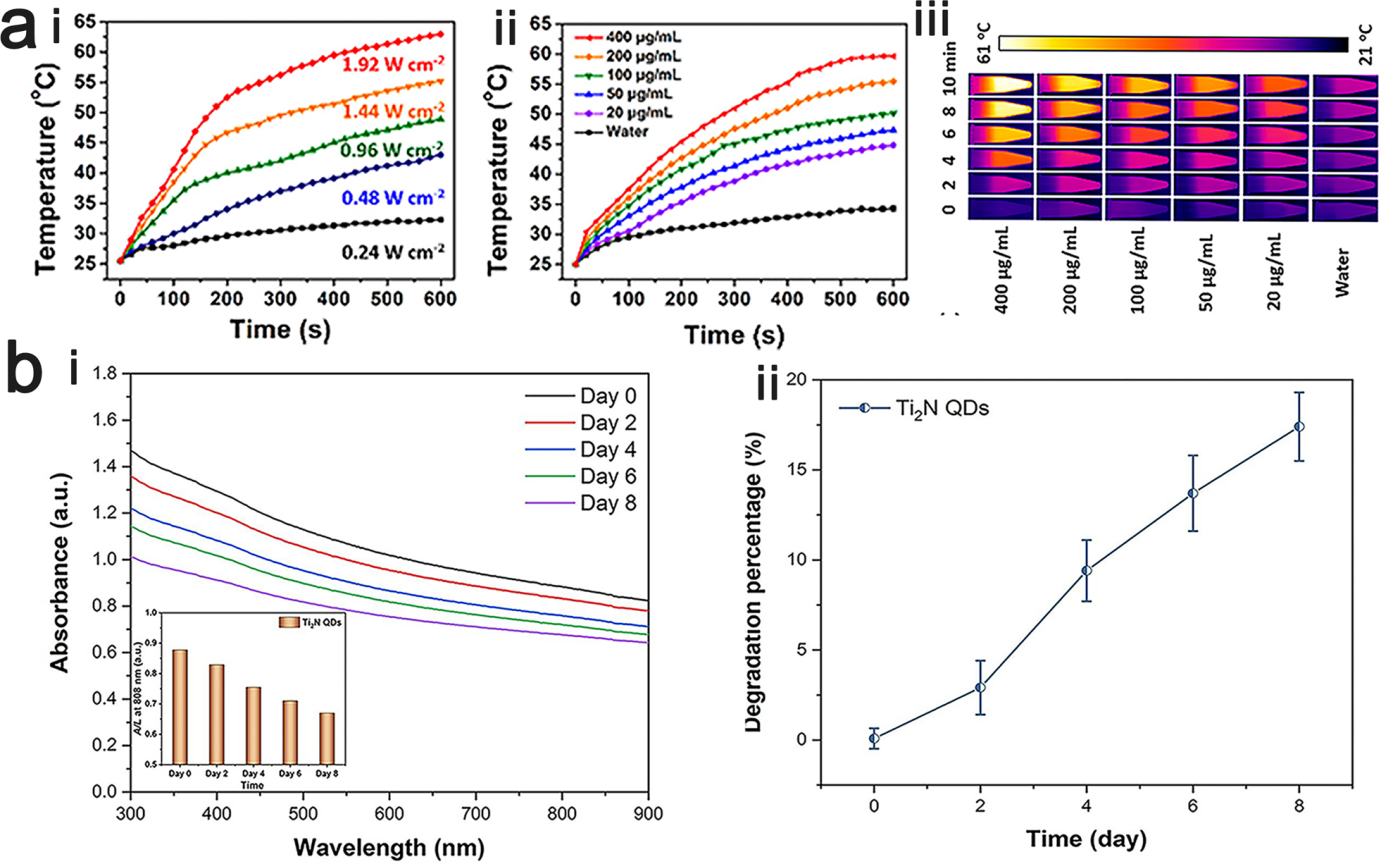
**a** (i) Photothermal heating curves of V_2_CT_x_-TAT@E_x_-RGD (V_2_CT_x_-TAT, 100 μg/mL) solution under 1064 nm (NIR-II) laser irradiation at different power densities. Photothermal heating curves (ii) and corresponding thermal images (iii) of V_2_CT_x_-TAT@E_x_-RGD solution under 1064 nm laser irradiation at different concentrations at a power density of 0.96 W cm^−2^. Reproduced with permission from Ref. [152], © American Chemical Society 2019. **b** Absorption spectra (absorption intensity at 808 nm (*A/L*) (i) and percentage degradation(ii) of Ti_2_NT_x_ quantum dots after degradation in water for 0, 2, 4, 6 and 8 days. Reproduced with permission from Ref. [150], © Elsevier 2020.

PTT has reduced side effects compared to traditional cancer treatment modalities due to the high spatiotemporal control of local heat. To overcome heat shock protein (HSP)-induced thermal resistance to achieve effective tumor tissue ablation, the temperature of PTT usually needs to exceed 50 °C, which may lead to thermal damage to normal organs near the tumor [151]. Moreover, it was shown that the second near-infrared (NIR-II) biological window (1000–1350 nm) is more favorable than the NIR-I biological window (750–1000 nm) for achieving deep tissue penetration [18]. Cao et al. proposed a strategy for cryogenic nuclear-targeted PTT in NIR-II region-modifying small fluorescent V_2_CT_x_ quantum dots with good photothermal effects in the NIR-II region with TAT peptide and constructed a V_2_CT_x_-TAT@E_x_-RGD multifunctional thermal therapy platform by RGD modification (Fig. 13a) [152].

**Fig. 14:**
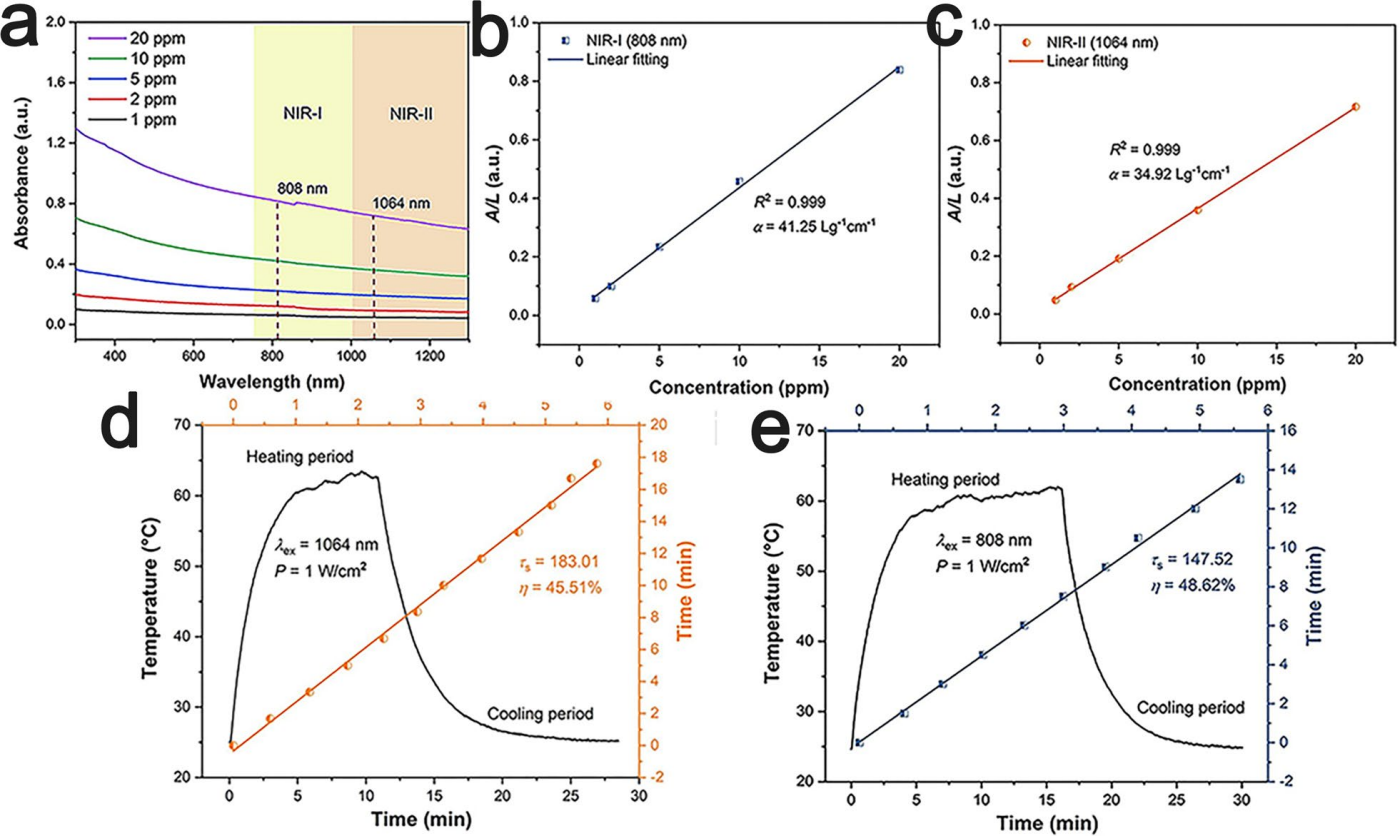
In vitro photothermal experiments. **a** Absorption spectra of Ti_2_NT_x_ quantum dots at different concentrations (NIR-I: 750–1000 nm, NIR-II: 1000–1350 nm). **b**, **c** Normalized absorbance intensities at 808 nm and 1064 nm for the characteristic cell length (*A/L*) of Ti_2_NT_x_ quantum dots. **d**, **e** Corresponding calculations of photothermal conversion efficiency. Reproduced with permission from Ref. [150], © Elsevier 2020

Moreover, Shao et al. studied the synthesis of nitride-based MXenes and Ti_2_NT_x_ quantum dots, which exhibited extremely high photothermal conversion efficiency in both the first and second near infrared (NIR) biological windows (NIR-I, 48.62% at 808 nm; NIR-II, 45.51% at 1064 nm) (Fig. 14) [150]. To our excitement, in addition to the good biocompatibility and photothermal therapeutic efficiency, Ti_2_NT_x_ quantum dots also show suitable degradation properties and excretion rate in vivo and can be smoothly excreted from the body after the PTT therapeutic effect has been exerted (Fig. 13b).

With significant photothermal conversion efficiency and strong absorption characteristics in the NIR range, MXenes has become an excellent PTT reagent for deep tissues. Moreover, different surface modifications and other strategies can significantly improve the photothermal properties and enhance tumour elimination.

#### Photodynamic therapy

Photodynamic therapy (PDT) is another very promising light therapy for the treatment of tumors. Photosensitizers (PSs) are an important factor in determining the effectiveness of PDT. By systemic or local administration, PS is allowed to accumulate at the tumor site. Then, photosensitizing molecules are activated in the presence of suitable wavelengths of light to form cytotoxic reactive oxygen species (ROSs) in the presence of endogenous molecular oxygen species, especially singlet-state oxygen, which eventually leads to cancer cell death [153, 154]. Due to their unique electronic structure and optoelectronic properties, MXene nanosheets are ideal materials for use as PDT photosensitizers. Since photosensitizer drugs have little toxicity until they are activated by external light, PDT can significantly reduce side effects and improve target specificity compared with conventional cancer treatment modalities such as radiotherapy and chemotherapy [155, 156].

In 2017, Liu et al. discovered the ability of Ti_3_C_2_T_x_ nanosheets to generate reactive oxygen species (ROS) under light and their potential as a novel photosensitizer for photodynamic therapy [136]. They used 1,3-diphenylisobenzofuran (DPBF) as a single-linear oxygen (1O_2_) detector and observed a significant decrease in the absorbance of DPBF under irradiation with 808 nm light, which indicated that 1O_2_ was produced by Ti_3_C_2_T_x_ nanosheets under irradiation with near-infrared light. The near-infrared laser-triggered generation of singlet oxygen by Ti_3_C_2_ nanosheets has led to its consideration as a novel photosensitizer for effective photodynamic therapy.

As research continued, it was found that in addition to Ti_3_C_2_T_x_, other MXenes have potential applications in the field of photodynamic therapy. Recently, Zhang et al. found that Mo_2_CT_x_ can also generate high temperature and ROSs under laser excitation, which can significantly induce apoptosis [157]. Encouragingly, by synthesizing 3D MXene with a honeycomb structure and anti-aggregation properties, Guo et al. found that 3D MXene has a higher ROS generation ability than Ti_3_C_2_T_x_ nanosheets [158].

Multiple studies have shown that MXene as a photosensitiser can produce reactive oxygen species under appropriate wavelengths of light which is important for cancer treatment. As research progresses, numbers of MXene is shown to have potential as photodynamic therapy.

#### Immunotherapy

Immunotherapy, which is a novel method for treating tumors, generates a durable antitumor response by enhancing or activating the patient's own immune system to achieve precise tumor treatment, targeted killing of tumor cells, and prevention of tumor recurrence and metastasis [159]. MXene-based materials are gradually showing unique advantages in the field of immunotherapy due to their excellent properties, such as high specific surface area, biocompatibility, and tumor-targeting accumulation. The advantages of MXene-based materials in the field of immunotherapy have gradually been demonstrated. However, it is usually difficult to achieve satisfactory results by immunotherapy alone, and a combination of immunotherapy with traditional nonimmunotherapy treatment modalities (e.g., chemotherapy, photothermal therapy, and photodynamic therapy) is usually utilized [160–163].

**Fig. 15:**
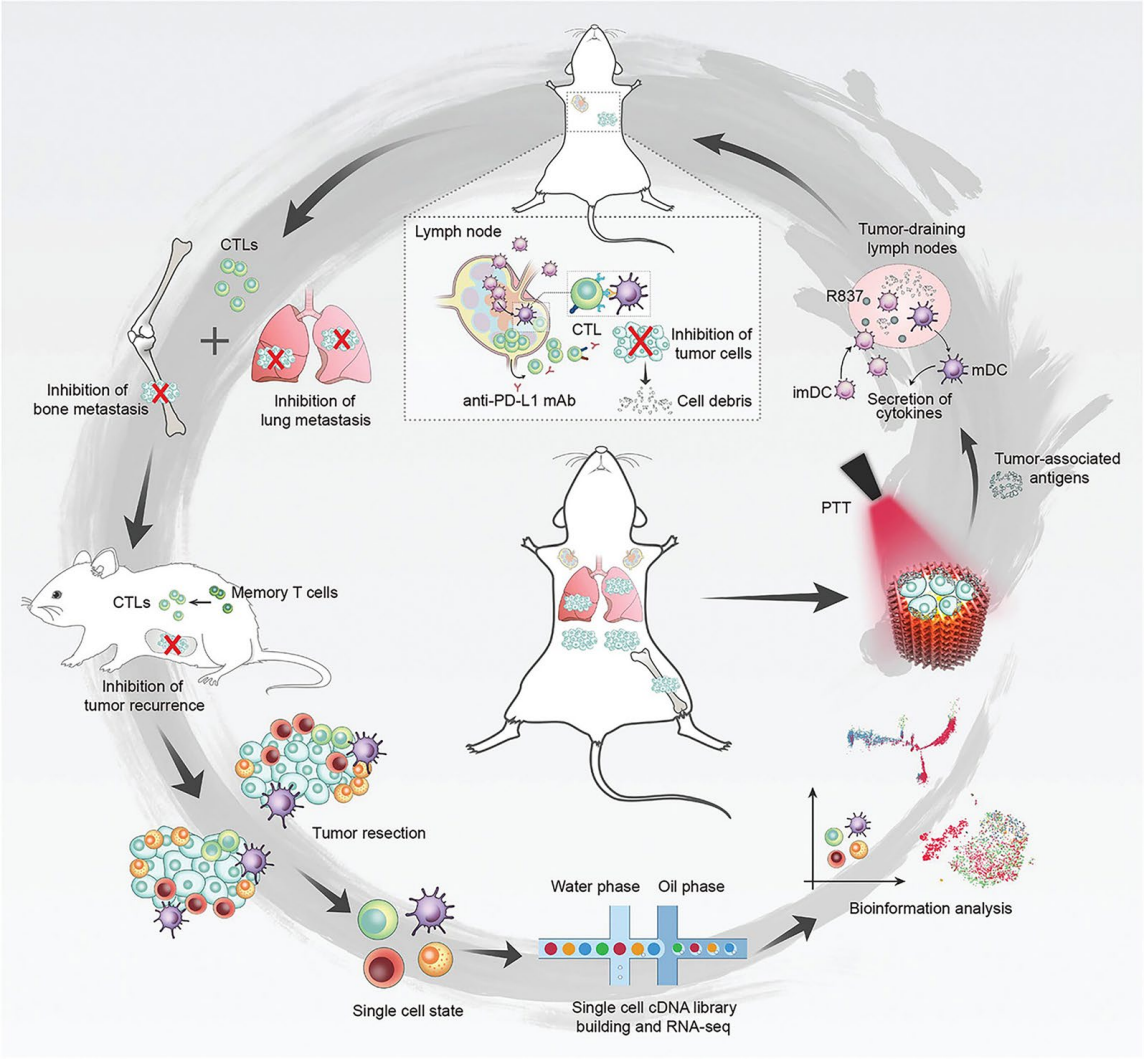
Schematic diagram of the mechanism of anti-tumor immune response induced by BG@NbSiR –based PTT plus anti-PD-L1 immunotherapy. Reproduced with permission from Ref. [161], © John Wiley and Sons 2021

**Fig. 16:**
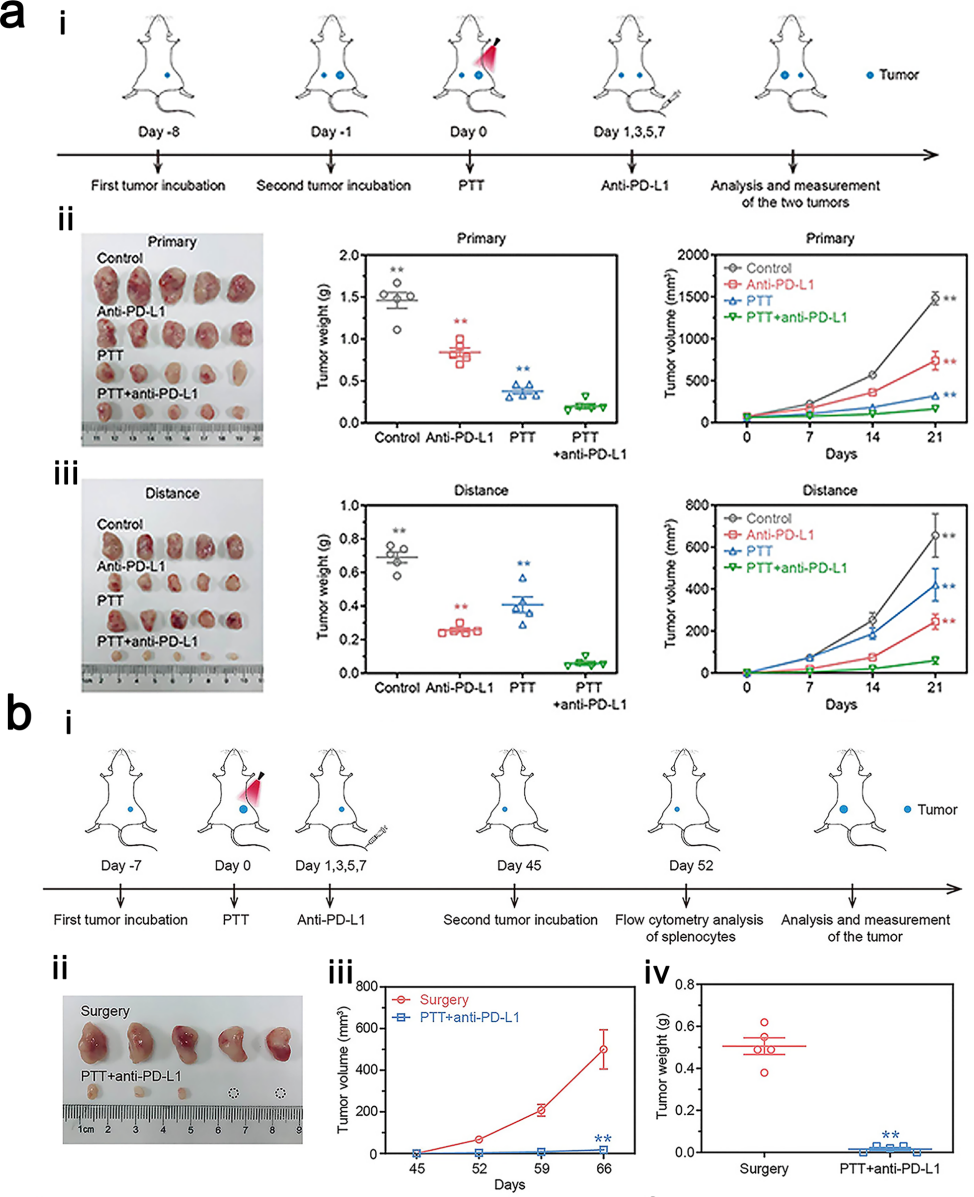
**a** Anti-tumor effects of BG@NbSiR-scaffold-based PTT plus anti-PD-L1 immunotherapy. (i) Scheme diagram of BG@NbSiR-scaffold-based PTT plus anti-PD-L1 combination therapy to suppress tumor progression at the distant orthotopic site. Tumor images, weight, and volume diagrams of (ii) the primary and (iii) the distant tumors after mice were sacrificed. **b** Long-term protection efficacy against tumor recurrence by BG@NbSiR scaffold-based PTT plus anti-PD-L1 immunotherapy. (i) Schematic illustration of BG@NbSiR-scaffold-based PTT plus anti-PD-L1 combination therapy to inhibit tumor recurrence (*n* = 5). Tumor (ii) images, (iii) volume, and (iv) weight diagrams of the rechallenged tumors after mice were sacrificed. Reproduced with permission from Ref. [161], © John Wiley and Sons 2021.

In 2020, Hao et al. designed and constructed a multifunctional niobium carbide (Nb_2_CT_x_) MXene-modified 3D printing biodegradable scaffold that was loaded with an immune adjuvant (R837) for the treatment of bone metastases of breast cancer. The prepared BG@NbSiR scaffold could induce tumor ablation by a thermal effect under the action of an 808 nm NIR laser. Following tumor ablation, tumor fragments were released in large quantities, which together with R837 could perform a vaccine-like function to promote dendritic cell (DC) recruitment/maturation and cytokine secretion, thereby activating an immune response to attack tumors (Fig. 15). In particular, combination therapy with PD-L1 checkpoint blockade protected the organism from breast cancer bone metastases by inducing DC recruitment/maturation at the tumor site and CTL infiltration, which awakened the immune system to eliminate both primary and metastatic tumors (Fig. 16a). In addition, the combination therapy established long-term protection by stimulating the host to produce immune memory, thereby effectively avoiding tumor recurrence (Fig. 16b) [161]. Meanwhile, the biodegradation products of the BG@NbSiR scaffold also promoted the subsequent bone regeneration process [161]. As another example of the application of Nb_2_CT_x_, Lu et al. constructed a Nb_2_C@PDA-R837@RBC smart nanoplatform with stronger PTT effects and enhanced immunotherapeutic effects that used polydopamine (PDA)-coated Nb_2_C nanosheets to load the immune adjuvant R837 and was coated with a red blood cell membrane (RBC) on the surface. After Nb_2_CT_x_@PDA-R837@RBC NPs induced effective ablation of primary tumor foci, TAAs and R837 were successfully released to stimulate dendritic cell (DC) maturation, thereby achieving the immunotherapeutic effects of killing primary tumor cells, inhibiting tumor cell growth and preventing tumor recurrence. In addition, coating an erythrocyte membrane on the nanoplatform can avoid excessive blood clearance and prolong blood circulation, thereby resulting in better biocompatibility[135]. In another application of (Ti_3_C_2_T_x_)MXene material, a nanocomposite drug delivery system (Ti_3_C_2_T_x_@Met@CP) was established by layer-by-layer adsorption of metformin (Met) and composite polysaccharide (CP) on the surfaces of Ti_3_C_2_T_x_ nanosheets to achieve a combination of PTT/PDT/chemotherapy/immunotherapy for complete tumor eradication and effective inhibition of tumor recurrence and metastasis. CP is a novel immunomodulator that mixes lentinan, pachymaran and tremella polysaccharides in optimal proportions. The modification of CP on the surfaces of Ti_3_C_2_T_x_ nanosheets improves its tumor site aggregation and biocompatibility and activates the immune function of the host [162]. In addition, a study prepared a Ti3C2-PEG-OVA-Mn^2+^ (TPOM) nanoplatform for PTT. The nanoplatform can release OVA and Mn^2+^ upon the irradiation of NIR laser, which simultaneously activated an anti-tumour adaptive immune response and natural immunity of the STING pathway, boosting DC maturation and increasing CTL infiltration validated by in vitro*/vivo* experiments [163].

In addition to being used as drug carriers of immune adjuvants or immunomodulators for combination immunotherapy, the immunomodulatory effect of MXenes may enable them to be used for immunotherapy directly [160, 164]. Recently, Rafieerad et al. experimentally found that Ti_3_C_2_T_x_ quantum dots can produce immunomodulatory effects in purified T cell populations without dedicated antigen-presenting cells (APCs), which enables them to independently suppress inflammatory activation, thereby demonstrating their potential as novel immunomodulatory platforms [160].

#### Synergistic therapy

Compared to the application of MXene alone in one area of treatment or diagnosis, researchers are more interested in developing its effective synergistic therapeutic effects with multiple treatment modalities. Combining PTT and PDT with other therapeutic diagnostic modalities synergistically can often produce unexpected results, such as combining PTT with PDT [165], PTT with SDT [166] or PTT with conventional chemotherapy and PDT or PTT with drug delivery [167]. Qun et al. found that the synergistic effect of Mo_2_C-mediated PDT/PTT (induction of apoptosis) was significantly better than that of PDT or PTT alone by using the ROS buster NaN_3_ to eradicate ROSs and using Mo_2_C-mediated phototherapy in an ice bath to eliminate the effects of PDT and PTT [157]. In 2017, Liu et al. developed a therapeutic nanoplatform for synergistic PTT/PDT/chemotherapy based on the advantages of Ti_3_C_2_T_x_ nanosheets with tumor-specific accumulation, stimulated-responsive drug release, and excellent biocompatibility, which was proven to have excellent tumor ablation effects in ex vivo experiments [136].

Of course, in addition to cancer treatment, drug delivery in combination with PDT or PTT can also be used for imaging, thereby leading to the development of new nanoplatforms for therapeutic and diagnostic applications. Based on the powerful X-ray attenuation ability and high NIR absorbance of tantalum carbide (Ta_4_C_3_T_x_) nanosheets, Han et al. constructed a novel multifunctional nanosystem for dual-mode CT and PA imaging of living tumors and efficient in vivo photothermal ablation of mouse transplanted tumors [71].

### Portable and wearable devices

In recent years, with the increased demand for flexible, efficient and high-performance devices, wearable, portable and highly sensitive devices have attracted much research interest in monitoring human health and human activities, among other applications.

**Fig. 17:**
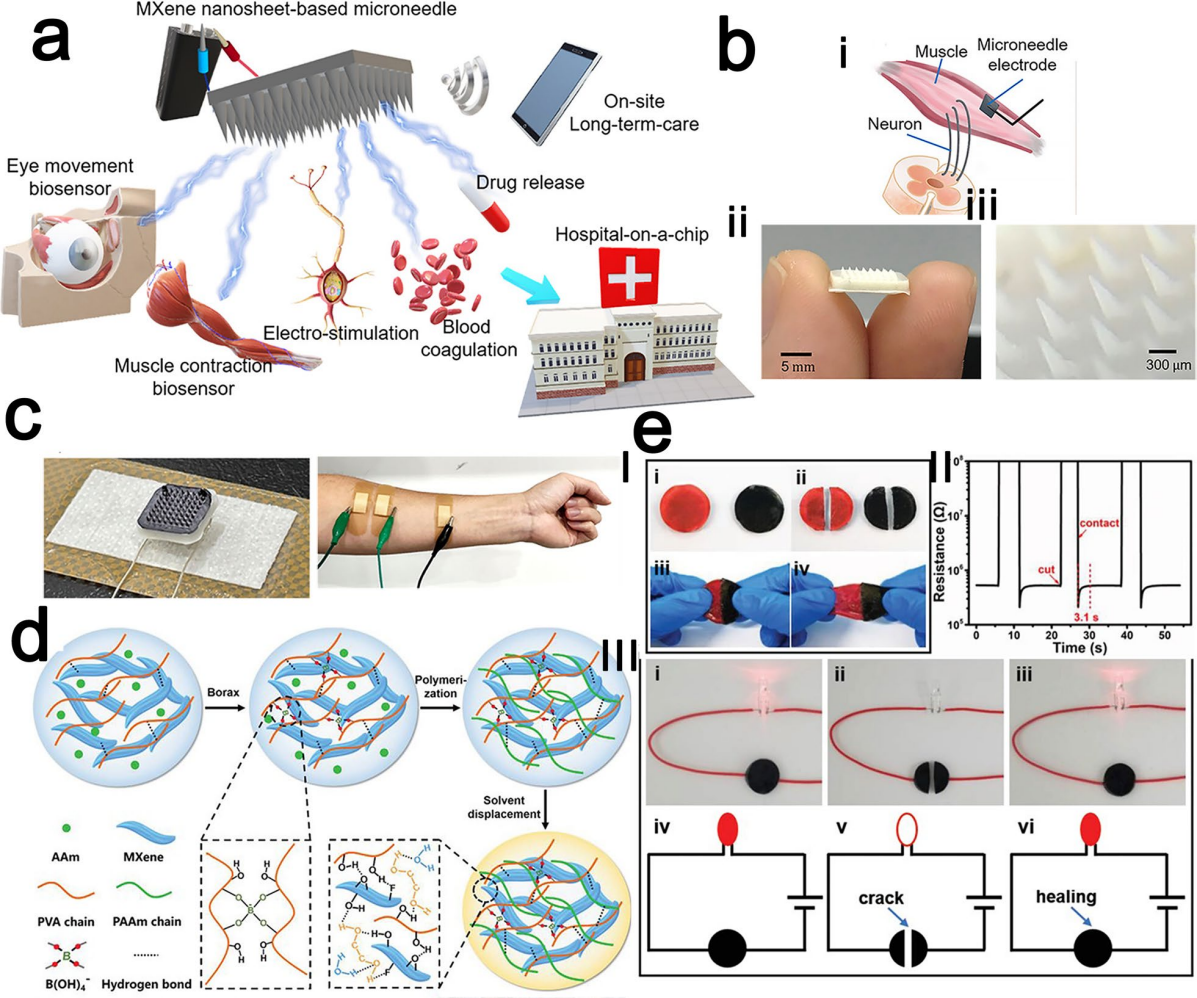
**a** Schematic diagram of the "hospital-on-a-chip" concept. **b** (i) Schematic representation of bioelectrical signal transmission from the neuron to the electrode or vice versa. (ii) Photograph of a PLA microneedle. (iii) Image of a close look of the tiny needles. **c** Photographs of wearable microneedle electrodes. Reproduced with permission from Ref. [171], © American Chemical Society 2021. **d** Schematic illustration of the fabrication of a conductive, anti-freezing, and self-healing conductive MXene nanocomposite organohydrogel (MNOH). **e** The experiment of self-healing. (I)The self-healing behavior between the original MNOH (black) and the MNOH dyed with rhodamine (red). (II) Time evolution of the healable process for the conductive MNOH by the real-time resistance measurements. (III) A circuit comprising MNOH in series with a red LED indicator: (i) original, (ii) completely bifurcated, (iii) self-healed, and (iv–vi) the corresponding schematic diagrams of the circuit. Reproduced with permission from Ref. [172], © John Wiley and Sons 2021

To date, most research has focused on the development of sensors. In 2017, Gao et al. first reported a highly flexible and sensitive piezoresistive sensor that is based on Ti_3_C_2_T_x_ for detecting subtle human activities and other weak pressures [168]. Large changes in the interlayer distance of Ti_3_C_2_ under external pressure were detected by in situ transmission electron microscopy, which demonstrated the basic operating mechanism of the piezoresistive sensor. The resultant sensor showed high sensitivity (gauge factor ~ 180.1), excellent flexibility, fast response (< 30 ms) and extraordinarily reversible compressibility (over 4000 times). Lei et al. developed a biosensor for sweat analysis that was made from a novel MXene/Prussian blue (Ti_3_C_2_T_x_/PB) composite, which provided a promising means for noninvasive monitoring of biomarkers. The unique modular and solid–liquid–air three-phase interface design enabled durable and sensitive detection of biomarkers (e.g., glucose and lactate) in sweat with a typical electrochemical sensitivity of 35.3 µA·mm^−1^·cm^−2^ for glucose and 11.4 µA·mm ^−1^·cm^−2^ for lactate [169]. In another study, Ti_3_C_2_T_x_ was also used to prepare a microfluidic wearable impedimetric immunosensor for the noninvasive detection of sweat [170]. Recently, Yang et al. developed a wearable, multifunctional microneedle system using highly stable MXene nanosheets to build a "hospital-on-a-chip" system with effective diagnostic and therapeutic applications [171]. The system consists of integrated microchip biosensors with real-time biosensing, electrical stimulation, and drug release capabilities (Fig. 17a, b and c). MXene has also been compounded with conductive materials such as conductive hydrogels with good stretchability [172, 173], flexible supercapacitor (FSC) [174] to prepare wearable sensors. Wan et al. introduced MXene sheets with excellent electrical conductivity into a hydrogel polymer that was composed of polyacrylamide (PAAM) and polyvinyl alcohol (PVA) to prepare a wearable electronic sensor with antifreeze properties, long-lasting moisture retention properties, and self-healing ability [172] (Fig. 17d and e).

Recently, researchers have also taken full advantage of the properties of MXenes, such as pressure sensing, temperature sensing, and energy storage, and compounded MXenes with other materials to discover the application potential of MXenes in human physiological activity monitoring, health care, personalized medicine, artificial skin, and human–computer interaction, among other areas.

In smart textiles, researchers have exploited the excellent electrical and thermal conductivity of MXenes by interacting them with various polar polymer textiles for the preparation of multifunctional textiles. In 2018, Zhang et al. prepared the first highly conductive hydrophobic textiles with excellent electromagnetic interference (EMI) shielding efficiency and Joule heating properties by depositing in situ polymerized polypyrrole (PPy)-modified MXene flakes onto poly(ethylene terephthalate) textiles followed by a silicone coating [175]. Gao et al. developed a superhydrophobic and breathable elastic MXene-based smart textile device with a multicore-shell structure. They modified MXene sheets on the surface of PDA-modified fibers by van der Waals forces and hydrogen bonding and constructed a multicore-shell structure on a polydimethylsiloxane (PDMS) coating. The prepared MXene-based smart textile devices showed not only good superhydrophobic permeability and mechanical durability and excellent photothermal response and electrothermal response but also ideal strain sensing properties and excellent temperature sensing behavior, which demonstrates the potential for applications in next-generation all-in-one wearable electronics, including for human motion and temperature monitoring, health care and personal thermal management [7].

Electronic skin with recognition and sensing capabilities beyond those of biological skin has vital applications in intelligent prosthetics, humanoid robotics and health monitoring. Taking advantage of MXene's adjustable surface termination, rich chemical properties and excellent electrical conductivity, MXene can be compounded with other materials to create multifunctional monitoring sensors. In 2022, Shen et al. prepared a flexible bimodal electronic skin based on a bionic chitosan/MXene (CTS/MX) hybrid membrane using chitosan (CTS) as a bridging agent. The electronic skin can also recognize pulses, sound signals, breathing rates and other human life activities, and the skin utilizes new strategies for developing the next generation of robust systems based on multifunctional wearable sensors. The prepared CTS/MX thin film electronic skin enables fast recognition of pressure and continuous monitoring of humidity. Moreover, the flexible bimodal e-skin has shown satisfactory results in recognizing pulses, sound signals, breathing rates and other human life activities. The excellent properties of the skin offer broad application prospects for its application in intelligent flexible systems such as electronic skins, bionic robots, biomedical devices and haptic feedback systems [176].

Husam et al. developed a self-charging power unit for the first time by integrating a triboelectric nanogenerator with MXene-based microsupercapacitors. This device could simultaneously and effectively collect and store mechanical energy of human biomechanical motions for powering electronics using skin as the contact, thereby opening up new possibilities for wearable/implantable sensor networks [177].

### Antibacterial agents

Over the past few decades, antibiotic-resistant bacterial infections have progressively become a major public health threat as bacteria become more resistant to conventional antibiotics, coupled with the increasing difficulty of discovering new effective antibiotics. Therefore, the development of novel antimicrobial agents for fighting drug-resistant bacterial infections is currently a major research objective in this field. Two-dimensional nanomaterials, which are represented by graphene and MoS_2_, have offered numerous excellent opportunities for the study of highly effective antimicrobial agents due to their unique two-dimensional structures. According to relevant reports, novel 2D nanoparticles show higher membrane permeability than antibiotics. The generation of reactive oxygen species (ROSs) and free radicals, enhanced oxidative stress, damage to genomic DNA, damage to cellular structural integrity and physical damage to cell membranes due to the sharp edges of 2D materials have been reported as the main antibacterial mechanisms of 2D nanoparticles [178–182]. MXene-based materials, which are characterized by a large specific surface area, feasible chemical manipulation and functionalization, and the potential to load various antimicrobial functional groups, are considered to be high-potential antimicrobial agents.

**Fig. 18:**
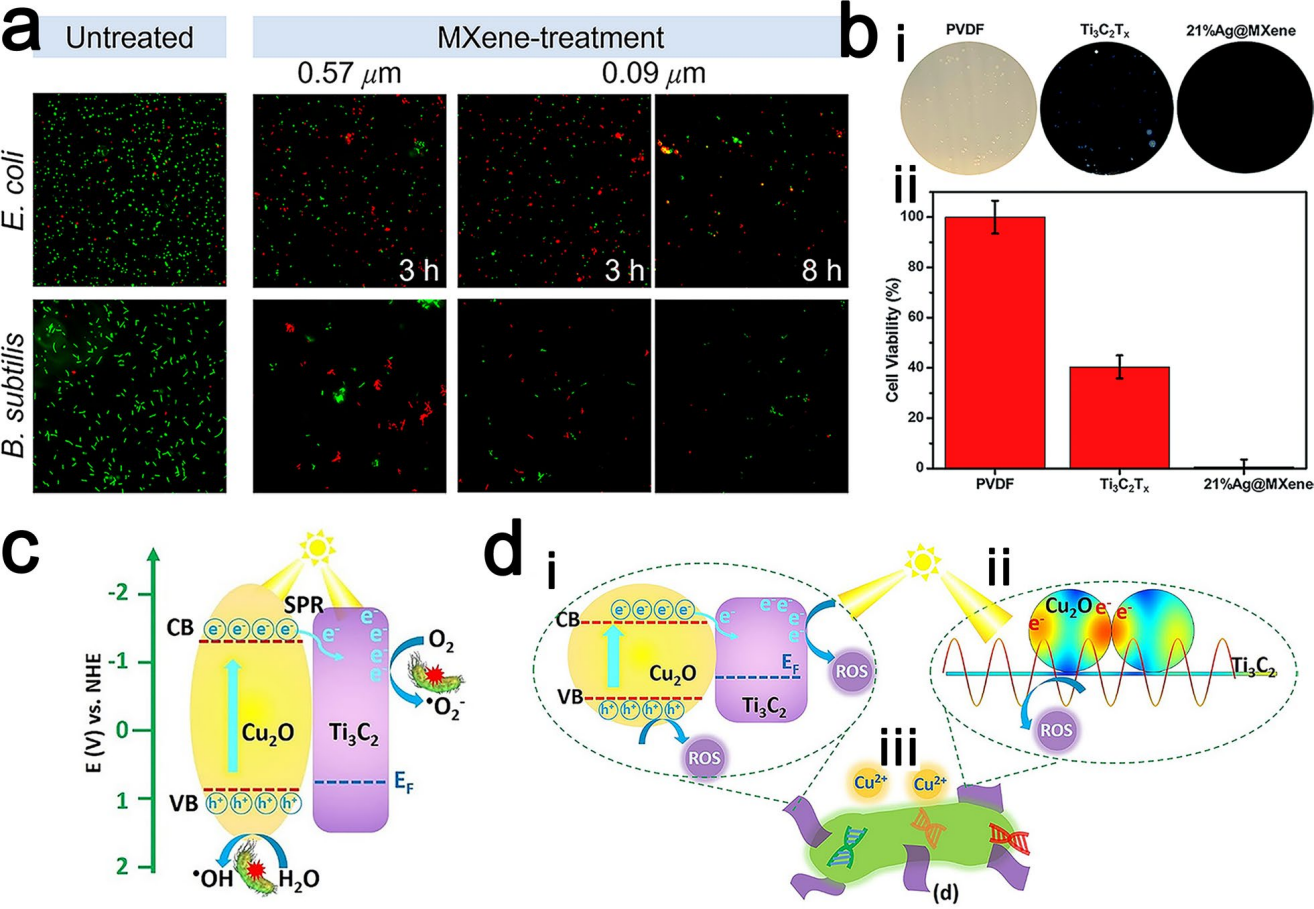
**a** Size dependence and exposure time dependence of antimicrobial activity of MXenes: Fluorescence imaging analysis was performed after treatment of Bacillus subtilis and Escherichia coli with 100-μg/ml MXene nanosheets of 0.09 and 0.57 μm size. Reproduced with permission from Ref. [183], © American Chemical Society 2018. **b** Antibacterial activity of PVDF (control), MXene (Ti_3_C_2_T_x_), and 21% Ag@MXene membranes. Reproduced with permission from Ref. [188], © Royal Society of Chemistry 2020. **c** Schematic of enhancement mechanism of synergistic antibacterial ability of Cu_2_O/MXene. Reproduced with permission from Ref. [189], © Elsevier 2020. **d** Schematic diagram of the synergistic antibacterial mechanism of Cu_2_O/MXene. Reproduced with permission from Ref. [189], © Elsevier 2020

In 2016, Rasool and Mahmoud et al. reported that Ti_3_C_2_T_x_ exhibited antibacterial behavior in colloidal suspensions, which was the first observation after graphene oxide in which Ti_3_C_2_T_x_ was found to act as an antibacterial agent [20]. By examining the inhibition effects of three materials, namely, Ti_3_AlC_2_T_x_(MAX), ML-MXene, and delaminated Ti_3_C_2_T_x_ nanosheets, which were tested by the colony counting method, it was found that all three materials had inhibitory effects on both E. coli and B. subtilis. In particular, a Ti_3_C_2_T_x_ colloidal solution led to 97.70 ± 2.87% and 97.04 ± 2.91% viability losses of E. coli and B. subtilis. Subsequently, they found that the antibacterial activity of Ti_3_C_2_T_x_ was dose-dependent by measuring the growth curves and cell viability of bacteria in Ti_3_C_2_T_x_ colloidal solutions of various concentrations. When the concentration of Ti_3_C_2_T_x_ was 200 μg/mL, the bacterial inhibition rate increased to more than 99%. Moreover, comparing the antibacterial activities of Ti_3_C_2_T_x_ and GO, the Ti_3_C_2_T_x_ had higher cell inactivation than GO. By LDH release analysis, SEM and TEM images and glutathione oxidation analysis, the following antibacterial mechanism of Ti_3_C_2_T_x_ nanosheets was proposed: Ti_3_C_2_T_x_ adsorbs on the cell surface, thereby leading to cell membrane rupture, and eventually the cells are damaged and die. Shamsabadi et al. investigated the antibacterial performances of MXene nanosheets of various lateral sizes (0.09, 0.35, 0.57 and 4.40 μm) against Bacillus subtilis and Escherichia coli bacteria using flow cytometry and fluorescence imaging techniques, and they confirmed both the size-dependent and exposure-time-dependent antibacterial performances of MXenes, which was the first study of the main antimicrobial mode of action of MXenes [183]. In addition, they used a broth microdilution assay for the first time to determine the correlation between the interaction between MXene nanosheets and bacterial cells and the antimicrobial performance of the nanosheets. They discovered that the sharp edges of the MXene nanosheets damaged the bacterial cell walls significantly, thereby leading to the release of bacterial DNA and consequently the dispersion of the bacteria (Fig. 18a). Making full use of the antibacterial properties of MXenes, Mayerberger et al. functionalized electrospun CS nanofiber mats with Ti_3_C_2_T_x_ sheets for the first time to prepare a flexible bandage material with remarkable antibacterial properties, which led to a cell reduction rate of 95% against gram-negative bacteria (Escherichia coli) and 62% against gram-positive bacteria (Staphylococcus aureus) [184]. A series of studies are currently underway to compound MXene with materials such as chitosan [185], electrospun poly (polycaprolactone) [186] and chitin [187] for the preparation of multifunctional composite films with excellent antibacterial properties, excellent biocompatibility and promotion of wound healing.

In recent years, various studies [20, 183, 184, 188–190] have demonstrated the excellent antimicrobial activity of MXenes. However, recently, it has been shown that MXenes can be modified by using nanoparticles of metals and metal oxides with antimicrobial activity (e.g., silver, zinc, and copper) [191, 192] to further enhance their antimicrobial properties. In 2018, Pandey et al. prepared Ag@MXene composite nanopore membranes by self-reduction of AgNO_3_ to generate AgNPs on the surfaces of MXene nanosheets [188]. In this experiment, E. coli was placed on PVDF (control), MXene and 21% Ag@MXene composite membranes and incubated at 35 °C for 24 h. According to the results, the 21% Ag@MXene composite membrane inhibited the growth of E. coli up to 99%, while the inhibition rate of the MXene membrane against E. coli was approximately 60% (Fig. 18b). In 2020, a cuprous oxide-anchored MXene nanosheet showed good antibacterial activity against Staphylococcus aureus and Pseudomonas aeruginosa bacteria with inhibition rates of 97.04% and 95.59%, respectively [189]. The Cu_2_O-anchored MXene nanosheets showed greatly enhanced antibacterial activity compared to the original MXene nanosheets through the synergistic effects of MXene acceleration of photoelectron transfer, Cu_2_O antibacterial activity and photocatalysis, generation of reactive oxygen species (ROSs), and ionophore resonance (Fig. 18c, d). In the future, it is possible that the development of new MXene compositions will lead to the discovery of alternatives with more antimicrobial potential. On the other hand, the high-photothermal conversion efficiency in the near-infrared radiation (NIR) biological window imparts or enhances the antimicrobial ability of MXene [193–196]. The strong antimicrobial properties make MXene a new multifunctional wound dressing. Simultaneously, the mild photothermal action is conducive to the promotion of cell proliferation and angiogenesis, facilitating the repair and remodeling of damaged tissues [197]. Herein, the development of MXene based wound dressings has attracted more and more attention. Gold nanoparticles (AuNPs) exhibit various unique properties including low toxicity, photothermal effects and polyvalent effects, as well as accelerating the keratinocytes and fibroblasts migration to speed up the skin repair. Xu et al. prepared a chitin/MXene composite sponge by incorporating MXene-based nanomaterials with gold nanoparticles (AuNPs) into the network of chitin sponge. The prepared composite sponges showed predominant antibacterial activity through the synergy between the capture and the photothermal effects, and promoted normal skin cell migration to heal the infected wound [194]. Mo et al. successfully constructed an antibacterial nanofibrous membrane (MXene-AMX-PVA nanofibrous membrane) by mixing amoxicillin (AMX), MXene and polyvinyl alcohol (PVA) for the treatment of bacterially infected skin wounds. Under lower power density NIR irradiation, the hyperthermia generated by MXene inhibited the bacterial proliferation and accelerated AMX release, effectively enhancing the healing rate of bacterially infected wounds [195].

Further research is required to develop new MXene compositions, and it is possible to discover alternatives with more antimicrobial potential. What’s more, additional studies of the antibacterial mechanism of MXene is important to promote the application of MXene as an antibacterial agent.

### Implants

Although medical implants are now widely used in clinical treatment, various problems remain to be solved, such as immune reaction, postoperative infection, poor healing, and tumor regeneration. MXenes can be used as a surface coating on implants to enhance and toughen implants and even significantly reduce the probability of tumor recurrence and bacterial infection because of their excellent biocompatibility, biodegradability and antimicrobial activity [24, 198–201]. To tackle the challenges of tumor recurrence and bacterial infection problems with conventional treatments for osteosarcoma, Xie et al. developed a novel multifunctional implant (Sp@MXGelMA) that consists of MXene nanosheets, gelatin methacrylate (GelMA), and bioinert sulfonated polyetheretherketone (SP) [23]. Through in vitro and in vivo experiments, it was demonstrated that the Sp@MX-TOB/GelMA implant has enhanced cytocompatibility, osteogenic commitment of preosteoblasts and osseointegration, which are highly favorable for the treatment of bone loss after osteosarcoma resection. Yin et al. introduced photonic-responsive 2D niobium carbide Nb_2_CT_x_ nanosheets into 3D-printed bone-mimetic scaffolds for osteosarcoma treatment [198]. Due to the special photonic response of the integrated 2D Nb_2_CT_x_ nanosheets in the second near-infrared (NIR-II) biological window with a high tissue penetration depth, it is highly efficient in killing bone cancer cells and effectively inhibits tumor regeneration. In addition, the biodegradation of 2D Nb_2_CT_x_-integrated 3D-printed scaffolds can significantly promote the neovascularization and migration of the defective area, thereby substantially facilitating osseous regeneration to repair larger bone defects.

In recent years, several two-dimensional materials, such as graphene, have been shown to expedite osteogenic differentiation of human bone marrow mesenchymal stem cells [202, 203]. MXenes, which are graphene analogs, have also been shown to enhance cell proliferation and osteogenic differentiation capacity [204]. In 2019, Zhang et al. conducted the first study of the application of Ti_3_C_2_T_x_ MXene films in bone tissue engineering and GBR treatment [201]. The good cytocompatibility and cell proliferation ability of Ti_3_C_2_T_x_ were demonstrated by cellular experiments. It was confirmed that Ti_3_C_2_T_x_ films show no significant inflammation or toxic side effects by the host tissue response to MXene films in vivo, thereby further confirming their safety in vivo. Moreover, the results of an alkaline phosphatase (ALP) assay and qRT–PCR of MXenes also showed that MXenes promoted early osteogenic differentiation of preosteogenic cells, which was also confirmed in rat calvarial defect model experiments. Recently, Shi et al. developed the ultra-thin 2D Mxenes and testified the role of few-layered Nb_2_C (FNC) in reducing inflammatory cytokine production and inhibiting osteoclastogenesis via ROS scavenging by Micro-CT, histological assessments, and UHMWPE particle-induced osteolysis models [205].

In addition, recent studies show that two-dimensional Ti_3_C_2_T_x_ MXene promote neural stem cells (NSCs) differentiation and electrophysiological maturation of neural circuits, providing a critical and promising direction for a line of evidence for using Ti_3_C_2_T_x_ MXene in neural interface or scaffold in stem cell therapy and nerve tissue engineering from morphology, physiology and functionality. NSCs cultured on Ti_3_C_2_T_x_ MXene films differentiated into neurons with higher efficiency and longer neurites, demonstrating their capability to promote NSCs maturation. Furthermore, Ti_3_C_2_T_x_ MXene had no appreciative effect on voltage-gated Na^+^ or K^+^ currents, but selectively increases the amplitude of voltage-gated Ca^2+^ currents, which could contribute to longer neurons that may contribute to the longer neurites, as well as the boosted spiking and subsequently enhanced synaptic transmission. Ti_3_C_2_T_x_ MXene enhances synaptic transmission by selectively increasing the frequency instead of the amplitude of synaptic events or the number synapses [206].

The good biocompatibility, physical degradability and antibacterial activity of MXenes provide favourable conditions for tissue regeneration and good therapeutic efficacy. Meanwhile, based on the tumour-killing effect of MXenes and the bone regeneration ability, MXenes research not only provides a new type of nanomaterial but also open a new direction for biomedical applications of MXenes.

### Others

**Fig. 19:**
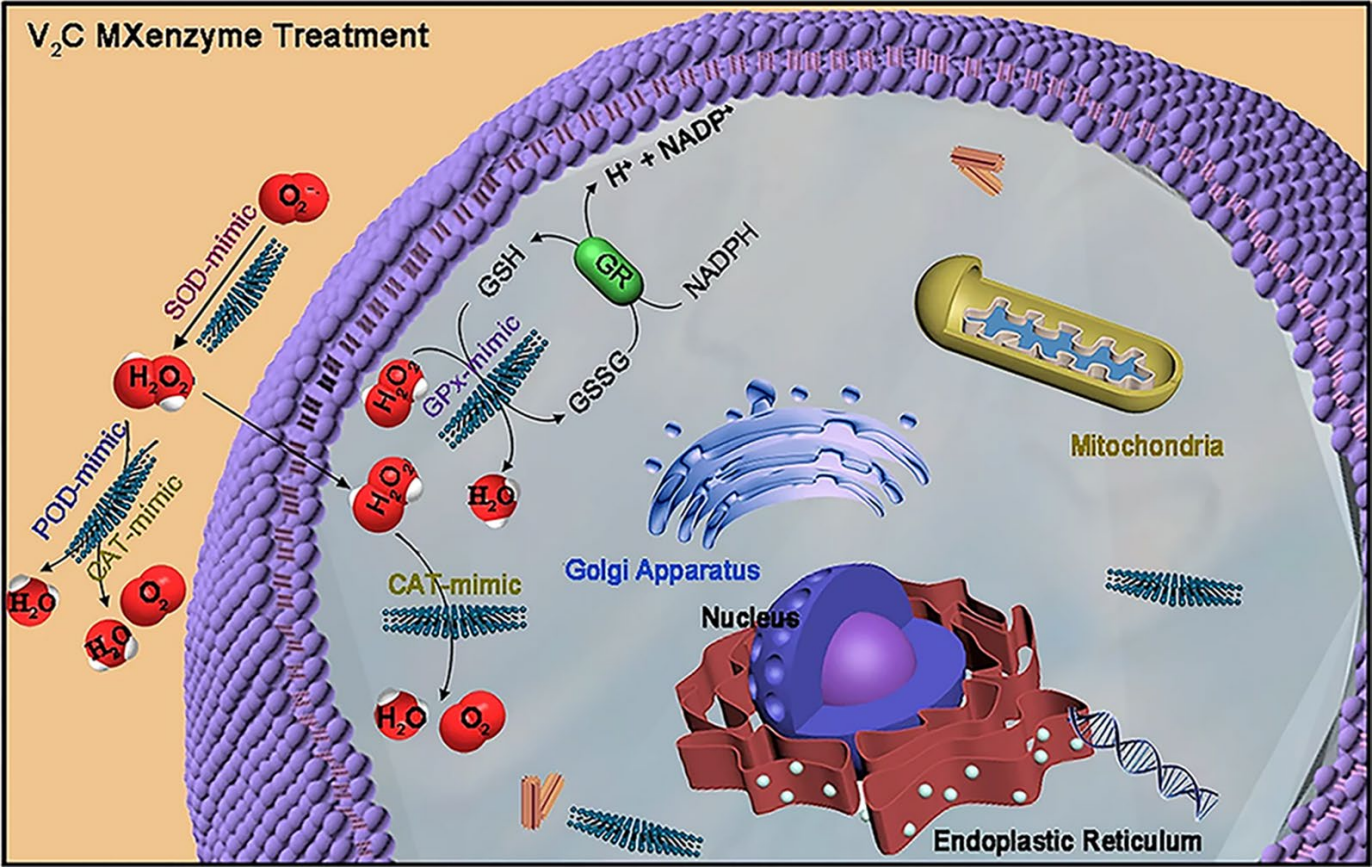
Schematic diagram of ROS scavenging by V_2_CT_x_ MXenzyme, which can effectively catalyze O_2_-· generation of H_2_O_2_ and O_2_, decomposition of H_2_O_2_ into O_2_ and H_2_O, and scavenging of ·OH. Reproduced with permission from Ref. [207], © Springer Nature 2021

In addition to the fields of application that are discussed above, it has recently been found that MXenes can also be applied as a nanoenzyme while scavenging excessive intracellular ROSs to realize powerful cytoprotective effects [207–209]. Reactive oxygen species (ROSs) are oxygen-containing chemically reactive molecules, which include singlet oxygen (1O_2_), superoxide anion radicals (O_2_-·), hydroxyl radicals (·OH), and hydrogen peroxide (H_2_O_2_). Two-dimensional V_2_C MX enzymes can effectively catalyze the transformation of O_2_-· into H_2_O_2_ and O_2_, which is produced by decomposition into O_2_ and H_2_O and scavenging of ·OH, thereby inhibiting the elevation of the intracellular ROS level and achieving smart cytoprotection against oxidative stress-induced inflammation and neurotoxicity (Fig. 19). These studies reveal new explorations into the applications of MXene nanomaterials, and in-depth research into the properties of MXenes is essential to expand their applications.

## Perspectives and summary

### Summary

Along with the development of nanomedical technology, MXenes are a promising new nanomaterial in the biomedical field. In this paper, we review the relevant advances in the application of MXenes and their derivatives in biomedicine in recent years. Starting from the preparation methods, we summarize and outline two main synthetic routes: a top-down route that is based on the direct exfoliation of multilayer bulk crystals and a bottom-up route that is based on the growth of 2D ordered structures by molecules/atoms. In addition, MXenes have great potential for surface modification and functionalization due to their rich surface capping functional groups (e.g., hydroxyl (–OH), fluorine (–F), and oxygen (–O)). Subsequently, due to the unique structural features, such as ultrathin atomic thickness and high specific surface area, and excellent physicochemical properties of MXenes, we reviewed and analyzed the applications of MXenes in biosensors, diagnosis, medical implants, antibacterial drugs and wearable devices. Overall, with large specific surface area, tunable optoelectronic properties and considerable biocompatibility, MXenes have been proven to be promising for a wide range of biomedical applications in biosensing, diagnosis and therapy, especially in immunotherapy and wearable devices, which are hot research areas. It is believed that MXenes will occupy an extremely important position in the future biomedical field with gradual research.

### Opportunities and challenges

Compared with traditional organic materials, inorganic 2D MXene nanomaterials show great potential for biomedical applications such as biosensors, drug delivery, and bioimaging due to their unique physicochemical properties, good biocompatibility, and easy functionalization, but they still face many challenges in clinical translation.

The first major challenge in the clinical translation of 2D MXene nanomaterials is their potential nontargeted toxicity. To translate these 2D MXene nanomaterials into clinical applications, it is not enough to continuously enhance their physicochemical properties (e.g., photothermal conversion efficiency and laser triggering); more importantly, their toxicity and biocompatibility must be evaluated to fully characterize their safety during clinical translation. Although in vivo and in vitro studies have been performed on the biocompatibility of MXenes and have shown short-term biocompatibility, these studies have generally been limited to short-term experiments. However, systematic evaluation of the long-term safety of MXene-based materials is essential for further expanding the practical application of these materials in the biomedical field. A recent study suggested that while the accumulation of Ti_3_C_2_T_x_ in the uterus barely affected the reproductive capacity of female mice, the neurotoxic effect on the offspring mice was evident. It is suggested that future studies should pay more attention to the long-term effects of nanomaterial exposure, including the health of adult offspring, especially neurodevelopment, rather than being limited to short-term effects, such as pregnancy outcomes [210]. The cellular uptake behavior, cytotoxicity mechanisms [211], immunogenicity, biodistribution, and factors that may affect the toxicity of MXene-based materials need to be further investigated, which will facilitate the development of strategies for modulating their toxicity, which is the next step of research. It has been shown that the surface functionalization, size, dispersion state, chemical composition, solubility, and crystalline shape of a 2D material may affect its toxicity and biocompatibility [212]. A common strategy for reducing the toxicity of MXene materials is surface modification. For example, collagen-modified MXenes have been shown to enhance ROS production in cancer cells while reducing ROS generation due to oxidative stress in normal cells, thereby reducing the toxicity of MXenes to normal cells. Rozmysłowska-Wojciechowska et al. used collagen to modify the surfaces of MXenes using the interaction between the MXene surfaces and natural biomacromolecule collagen, which was verified to reduce the toxicity of the MXenes and improve cell survival in in vitro experiments [213].

Second, during the biomedical application of MXenes, many MXenes are needed to load therapeutic drugs or other auxiliary reagents. Considering the damage of drugs to normal cells and the effective drug concentration during the treatment, achieving controlled release of drugs is also crucial for the construction of drug carriers. To date, researchers have developed two main strategies for controlled drug release: pH induction and NIR induction [214, 215].

Third, the properties of MXenes (e.g., toxicity) can be more easily controlled by precise design of their composition, size, and surface functionalization. The bottom-up synthesis approach is more controllable than the top-down approach, which lacks size distribution and reproducibility control. Moreover, in addition to the study of flat films and quantum dots, the study of MXenes with other morphological features, such as nanotubes and nanocages, is also valuable. Of course, combining MXenes with other types of functional materials to form hybrid materials and fully combining the advantages of multiple materials are additional research directions for MXene biomedical applications [216].

Fourth, to further promote the clinical translation of MXenes in the biomedical field, more efforts should be made to increase the low yield of MXenes in scale-up production.

In conclusion, the future of MXene-based biomaterials in the biomedical field is promising, but the road to their clinical application is still very long and arduous. It is highly expected that this review will provide some inspiration to research scholars in various fields and advance the process of clinical translation of novel MXenes and related materials.

## Data Availability

Not applicable, please refer to the original references.
